# Assessing the Validity of Asthma Associations for Eight Candidate Genes and Age at Diagnosis Effects

**DOI:** 10.1371/journal.pone.0073157

**Published:** 2013-09-09

**Authors:** María Pino-Yanes, Almudena Corrales, José Cumplido, Paloma Poza, Inmaculada Sánchez-Machín, Anselmo Sánchez-Palacios, Javier Figueroa, Orlando Acosta-Fernández, Nisa Buset, José Carlos García-Robaina, Mariano Hernández, Jesús Villar, Teresa Carrillo, Carlos Flores

**Affiliations:** 1 Centro de Investigación Biomédica en Red de Enfermedades Respiratorias (CIBERES), Instituto de Salud Carlos III, Madrid, Spain; 2 Research Unit, Hospital Universitario Nuestra Señora de Candelaria, Santa Cruz de Tenerife, Spain; 3 Allergy Unit, Hospital Universitario Dr. Negrín, Las Palmas de Gran Canaria, Spain; 4 Allergy Management Unit, Hospital del Torax (Ofra), Santa Cruz de Tenerife, Spain; 5 Allergy Unit, Hospital Universitario Insular de Gran Canaria, Las Palmas de Gran Canaria, Spain; 6 Neumology Unit, Hospital Universitario de Canarias, San Cristóbal de La Laguna, Spain; 7 Research Unit, Hospital Universitario Dr. Negrin, Las Palmas de Gran Canaria, Spain; 8 Allergy Unit, Hospital Universitario Nuestra Señora de Candelaria, Santa Cruz de Tenerife, Spain; 9 Applied Genomics Group (G2A), Genetics Laboratory, Instituto Universitario de Enfermedades Tropicales y Salud Pública de Canarias, Universidad de La Laguna, San Cristóbal de La Laguna, Spain; 10 Keenan Research Center, St. Michael' Hospital, Toronto, Ontario, Canada; Centro Cardiologico Monzino IRCCS, Italy

## Abstract

**Background:**

Before the advent of genome-wide association studies (GWAS), *ADAM33, ADRB2, CD14, MS4A2* (alias *FCER1B*), *IL13, IL4, IL4R*, and *TNF* constituted the most replicated non-*HLA* candidate genes with asthma and related traits. However, except for the *IL13-IL4* region, none of these genes have been found in close proximity of genome-wide significant hits among GWAS for asthma or related traits. Here we aimed to assess the reproducibility of these asthma associations and to test if associations were more evident considering the effect of age at diagnosis.

**Methodology/Principal Findings:**

We systematically evaluated 286 common single nucleotide polymorphisms (SNPs) of these 8 genes in a sample of 1,865 unrelated Spanish individuals (606 asthmatics and 1,259 controls). We found that variants at *MS4A2*, *IL4R* and *ADAM33* genes demonstrated varying association effects with the age at diagnosis of asthma, with 10 SNPs showing study-wise significance after the multiple comparison adjustment. In addition, *in silico* replication with GWAS data supported the association of *IL4R*.

**Conclusions/Significance:**

Our results support the important role of *MS4A2*, *IL4R* and *ADAM33* genes in asthma and/or atopy susceptibility. However, additional studies in larger samples sets are needed to firmly implicate these genes in asthma susceptibility, and also to identify the causal variation underlying the associations found.

## Introduction

Asthma is a complex respiratory disease characterized by chronic inflammation of the airways and frequently associated with atopy, pulmonary obstruction and bronchial hyper-responsiveness against a diversity of stimulus [1]. Its prevalence varies widely among different populations around the world (1–18%) [2]. Familiar clustering [3], twin studies [4], and genetic studies [5], [6] support an important genetic component of the disease, with an estimated heritability of 60% [7].

Before the advent of genome-wide association studies (GWAS) [8], almost a thousand candidate-gene association studies for asthma and related traits were published [9]. Considering the gene as the unit of replication and using a broad definition for asthma, Ober & Hoffjan [5] elegantly summarized the accumulated evidence for candidate-gene association studies from the literature by assessing the consistency of findings [10]. This yielded a ranking of candidate genes based on the number of positive associations between any polymorphism and any asthma trait [5]. As a result, eight non-*HLA* genes were put forward among the most replicated (in >10 independent studies) and, therefore, these genes were suggested as firm candidates for asthma susceptibility [5]. Four of these genes were located in the linked region for asthma on chromosome 5q: interleukin (*IL*) 4 (*IL4*), *IL13*, *CD14* and the β2-adrenergic receptor (ADRB2); one gene was located in the linked region 6p21: the tumor necrosis factor (*TNF*); one was the first positionally cloned asthma gene, ADAM metallopeptidase domain 33 (*ADAM33*); another was the gene encoding the α chain of the IL-4 and IL-13 receptors (*IL4R*); and finally, the gene encoding the IgE Fc receptor beta-subunit (*MS4A2*, alias *FCER1B*). However, most of them were assessed in studies with limited sample sizes averaging ≈200 individuals per group [5], [11], and lacked of a systematical analysis in candidate-gene studies by surveying more than few variants (for example, by using tagging SNPs [tSNPs]). To date, despite the fact that more than ten GWAS of asthma have been published, none of these eight firm candidates have been replicated at genome wide significance, nor have been found in close proximity of GWAS hits, except for the *IL13-IL4* region [8], [12]–[21].

Asthma is clinically recognized as an amalgam of several distinct phenotypes [22], [23], which blur the complex genetic architecture underlying the disease susceptibility. Among these phenotypes, the age-at-onset of asthma could differentiate asthmatic groups, so that genetic variants might inconsistently associate with childhood and later-onset disease [17], [24], [25]. Motivated by this evidence, here we aimed to assess the reliability of asthma associations for the eight most replicated non-*HLA* asthma candidate genes and to explore whether effects of risk alleles varied with the disease age at diagnosis.

## Methods

### Ethics statement

This study was approved by the External Scientific Committee and Advisory Committee of Experts on ethical, economic, environmental, legal and social affairs at the National Bank and the Ethics Committee of Hospital Universitario NS de Candelaria and Hospital Universitario Doctor Negrín. Written informed consent was obtained from all subjects or appropriate surrogates on the behalf of participants under the age of 18.

### Study subjects

This study was conducted using a case-control design of 1,878 DNA samples from unrelated individuals, all reporting at least two generations of Spanish descent. Sample details have been described elsewhere [24]. In brief, cases included 607 asthmatic patients aged >5 years and diagnosed by physicians following the Global Initiative for Asthma (GINA) guidelines for diagnosis and classification of asthma severity (http://www.ginasthma.com). These samples were collected and characterized for allergic and asthmatic symptoms in Respiratory Medicine and Allergy Departments, as part of the Genetics of Asthma study (GOA) in the Spanish population. Among cases, atopy was defined by the evidence of allergic sensitization to known allergens, reflected by either a positive skin prick test [SPT] or the specific IgE to one or more known allergens in the serum. For simplicity, those cases that had asthma and also atopy will be referred as atopic asthmatics, although we ignored whether or not allergen exposures lead to the asthma symptoms of these patients. Further sample details can be found in Text S1 and in Table S1.

Control group consisted of 1,271 DNA samples from adults self-reporting no personal or familiar medical history of allergic or pulmonary diseases recruited from the Spanish National DNA Biobank. These were collected from branches of the National Blood Service from unrelated individuals residing in Spain. After signed informed consent, by means of personal interviews, each donor was asked to declare general health status, physic activity, commonly used transportation, nutrition habits, type of work and qualification, demographics, tobacco smoke, alcohol consumption, genealogical information, residence and mother tongue, and personal and familial history of diseases. See http://www.bancoadn.org for further information. In addition to the criteria of the Spanish National DNA Biobank to define healthy controls, we added three more criteria to select the controls for this study: 1) Self-reported Spanish ancestry based on having at least two generations of ancestors born in Spain; 2) Complete data on personal and familiar history of disease recorded in the questionnaire, smoking status, place of origin, and area of residence; 3) Absence of self-reported personal or familiar history of pulmonary or allergic disease. Further sample details can be found in Text S1 and in Table S1.

### Selection of tagging SNPs

Tagging SNPs (tSNPs) were selected by means of TagIT 3.03 [26], using available re-sequencing data from European samples from different sources (Table 1 and Table S2). The *IL13* and *IL4* genes, which lie in close proximity, were considered as a single region. Similarly, given the strong linkage disequilibrium (LD) between *LTA* and *TNF* genes [27], common variants of the *LTA* gene were also tagged and jointly analyzed with *TNF*. See Text S1 and Table S2 for further details.

**Table 1:** Summary information used for the selection of tagging SNPs (tSNPs) on the candidate genes.

Gene	Chr. (Mb)	Size (kb)	Covered region (kb)	Data sources	Selected tSNPs	Monomorphic	Final tSNPs	Final haplotype *r^2^*
*IL13–IL4*	5q31.1 (132.0)	12	29.0	SeattleSNPs^a^	10	0	10	1.00
*CD14*	5q31.1 (140.0)	2	7.0	Innate Immunity^b^	6	1	5	1.00
*ADRB2*	5q31 (148.2)	2	9.5	SeattleSNPs^a^	8	1	7	1.00
*TNF-LTA*	6p21.3 (31.5)	6	9.3	SeattleSNPs^a^	11	0	11	0.85
*MS4A2*	11q13 (59.9)	10	15.3	HapMap/ T1D^c^	7	0	7	0.97
*IL4R*	16p12.1 (27.3)	51	56.0	SeattleSNPs^a^	21	0	21	0.92
*ADAM33*	20p13 (3.6)	14	15.2	EGP^d^	19	4	15	1.00
Total		97	141.3		82	6	76	

a The National Heart Lung and Blood Institute's (NHLBI) Programs for Genomic Applications (http://pga.gs.washington.edu).

b The Inate Immunity NHLBI Program for PGA (https://regepi.bwh.harvard.edu/IIPGA2).

c HapMap phase 2 (http://hapmap.ncbi.nlm.nih.gov) and data from 96 type 1 diabetes individuals [28].

dThe NIEHS Environmental Genome Project (http://egp.gs.washington.edu).

### Assessment of population stratification

To reduce the risk for false positives due to major population stratification effects, a total of 83 European ancestry informative markers (termed EuroAIMs) were determined in case and control samples. These EuroAIMs allowed to correct for major differences in Spanish populations due to the North African genetic influences observed in this population, with a mean value of 5–9% for mainland populations and 16–20% for Canary Islanders [28]. A principal component analysis (PCA) based on these genetic markers was used to derive the ancestry estimates in cases and controls as scores of the first principal component (PC1), by means of EIGENSOFT [29]. A full list of EuroAIMs used and the genotyping procedures have been detailed elsewhere [24], [28].

### Genotyping

Genotyping was conducted using the iPLEX® Gold assay on MassARRAY® system (Sequenom Inc., San Diego, CA) by the Spanish National Genotyping Center, Santiago de Compostela Node (CeGen, http://www.cegen.org). Briefly, iPLEX® assays were scanned by MALDI-TOF mass spectrometry and individual SNP genotype calls were automatically generated using Sequenom TYPER 3.4® software (Sequenom Inc.). Samples from the Coriell Institute for Medical Research (http://www.coriell.org) were included on each SpectroCHIP® (Sequenom Inc.) to test allele calling reliability samples of this platform. The SNPs that gave poor quality data on this platform were finally determined at the Hospital Universitario N. S. de Candelaria using either SNaPshot® Multiplex kit reactions (Applied Biosystems, Foster City, CA) or KASPar SNP Genotyping System assays (KBiosciences, Hertfordshire, UK). Genotyping was blind to the disease status and ≈6% of the samples were genotyped in duplicate to monitor genotyping quality. See Text S1 for further details.

### Statistical analysis

Clinical and demographical data were analyzed by means of the χ^2^-test and the Mann-Whitney U-test using R version 2.15 [30]. Departures from Hardy-Weinberg equilibrium (HWE) were evaluated separately for cases and controls using an exact test [31], by means of a custom script for STATISTICA (StatSoft Inc., Tulsa, OK) [32]. However, as deviations in cases have been considered a symptom of disease association [33]–[35], only those tSNPs deviating significantly from HWE in the control group were filtered out from further analyses (threshold *p-*value  = 7.0E-04 after considering the multiple comparisons performed). Individual tSNP associations were tested under an additive model by means of regression analysis with SNPassoc [36]. For that, PC1 scores were included as a covariate in regression models to adjust associations for population stratification, and allele effects were estimated as odds ratios (ORs) with 95% confidence intervals (CIs).

Additionally, MaCH 1.0 [37] was used to impute untyped SNPs with data from 380 European individuals deposited in The 1000 Genomes Project (1KGP), May 2011 version [38]. Association testing was performed using Mach2dat [37] adjusting for the PC1 scores. This analysis was conducted using allele dosages for those SNPs showing MAF≥10% and Rsq>0.3, ensuring that all SNPs considered for association testing were accurately imputed (with >90% of SNPs having Rsq>0.8, and with a mean Rsq across all imputed SNPs of 0.95 [IQR: 0.91–0.97]) (Table S3).

For each gene by separate, a conditional regression-based analysis was used to point out the independent association signals of each locus by including all SNPs associated at nominal significance. We then tested if association tests of the SNPs that represented nominal independent associations within each gene improved considering age-at-onset-varying effects, by implementing a sequential addition (SA) of cases [39]. For that, the age at diagnosis was utilized as a proxy for the age-at-onset of the disease, which was not recorded for most patients, and cases were grouped in categories of quartiles of age (14 [n = 155], 26 [n = 291], 39 [n = 427], and 82 years [n = 606]). The age at diagnosis cutoff obtained was next used to select a sub-sample of cases for which associations were tested again, both for tSNPs and imputed SNPs. LD patterns and regional association results were represented using LocusZoom 1.1 based on LD data from hg18 deposited by 1KGP [40].

To judge the significance of SNP associations in the context of the multiple comparisons performed, a false discovery rate (FDR) was calculated using QVALUE [41]. A FDR threshold of 5% (*p*-value ≤0.0012) was established to declare study-wise significance to limit the expected proportion of false positives incurred in the study when a particular individual SNP test was called significant. This was assessed considering altogether the *p*-values from all SNPs analyzed, both genotyped and imputed, the tests from the SA of cases to obtain the age cutoff at which the allele effects were largest, and all the comparisons performed (i.e. associations with asthma, atopic asthma, and age-of-onset before the cutoffs). Functional annotation of associated SNPs was carried out using the software HaploReg [42].

## Results

A total of 13 samples (1 case and 12 controls) were excluded from the analyses because of genotype quality (completion rate <90%). Out of the initial set of 82 tSNPs, 6 were found monomorphic (rs5744440, rs35684381, rs597040, rs8124875, rs614971 and rs17548816) by using both iPLEX® and an alternative genotyping method (see the Supplementary methods in Text S1). Only one tSNP (rs12361312 at *MS4A2*) deviated significantly from HWE expectations in the control group and was discarded from further analyses (Table S2). Therefore, a total of 75 tSNPs, which maintained an adequate coverage for all genes (*r^2^*≥0.85), and 211 imputed SNPs were considered for association studies in 1,865 samples (606 cases and 1,259 controls) (Table S3). The mean completion rate among the 75 tSNPs was 98.5% (P_25_–P_75_ = 98.7–100.0%), and the estimated overall genotype discordance rate among duplicated samples was 0.30% (95% CI  = 0.08%–1.09%).

Association testing revealed a total of 35 SNPs (16 tSNPs and 19 imputed) that were associated with either asthma or atopic asthma at nominal significance, although these were not considered significant in the context of the multiple comparisons (*p*-values >0.0012) (Table S3). Based on the premise that incorporating the age-at-onset in the analyses might increase the power to detect association [24], [25], we next used SA of asthma patients to estimate the age at diagnosis cutoff maximizing allele effects. For that, among the 35 SNPs that reached nominal significance, we first excluded the redundant SNPs from each gene using conditional logistic regressions for asthma or atopic asthma. We identified the following SNPs as the ones showing independent nominal associations: rs1800925 (−1112 C/T) in *IL13–IL4* (only for atopic asthma), rs2071590 in *LTA*-*TNF* (only for asthma), rs569108 (Gly237Glu) in *MS4A2*, and rs1805015 (Ser478Pro) in *IL4R* (both for asthma and atopic asthma) and rs2787095 in *ADAM33* (only for asthma) (data not shown). These 5 SNPs represented independent associations for each gene and coincidentally, these 5 SNPs had been associated with asthma or related traits in previous studies, but in this study we extended their association to a Southwestern European population with noticeable North African influences.

SA did not show any age at diagnosis cutoff that significantly maximized the association of rs1800925 (in *IL13–IL4*) with atopic asthma (lowest *p-perm*  = 0.063). In contrast, SA revealed allele effects peaking at the same age at diagnosis, 39 years (number of cases  = 427), for SNPs from *MS4A2* and *IL4R*: rs569108 in *MS4A2* (*p-perm*  = 0.005), and rs1805015 in *IL4R* (*p-perm*  = 0.001). However, SA revealed allele effects peaking at a different age at diagnosis for the SNPs from the other two genes: rs2071590 in *LTA*-*TNF* showing a maximum at 26 years (p-perm  = 0.002, number of cases  = 291), and rs2787095 in *ADAM33* with a maximum at 14 years (p-perm  = 3.0E–04, number of cases  = 155). The results obtained from the SA analyses using the quartiles of the distribution of the age at diagnosis were equivalent to those obtained using it as a continuous variable (data not shown).

**Figure 1:**
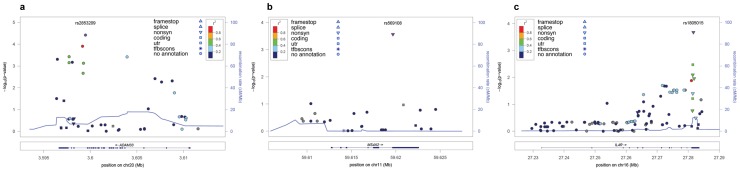
*P*-values of association by chromosome position with A) asthma ≤14years for *ADAM33*, B) asthma ≤39 years for *MS4A2* and, C) asthma ≤39 years for *IL4R*. ***P*-values are expressed in –log_10_ scale.** The SNP number shown on the plot denotes the result for the most significant SNP for each gene and the results for the remaining were color coded to reflect their LD with this SNP based on pairwise *r* ^2^ values from the 1KGP. Estimated recombination rates (from 1KGP) were also plotted on the right axis to reflect the local LD structure.

Testing associations on the sub-sample of cases with the age at diagnosis of asthma before the maxima determined by SA for each gene revealed 18 additional SNPs reaching nominal significance (Table S3). Five of these SNPs (0.013≤ p-value ≤0.050) were located in *IL4R* gene and only one of them constituted a positive finding in previous studies. After conditioning these new associations from IL4R to the SNP rs1805015, only one SNP showed independent association (rs3024676, p-value  = 0.021). The remaining 13 were all SNPs from *ADAM33* (3.8E-5≤ p-value ≤0.039), and 6 of them have been associated in at least one previous study (Table S3 and Figure 1). After adjusting the association of these 13 SNPs in ADAM33 that emerged with the age at diagnosis cutoff for the SNP rs2787095, 7 SNPs (rs2787093, rs628965, rs628977, rs630712, rs598418, rs2853209, and rs603112) resulted independently associated from this SNP (0.012≤ p-value ≤0.048). Therefore, the advantages of taking into account the age at diagnosis varying effects for replication studies in asthma were clearly evidenced in *ADAM33,* a gene for which SNP-level replications are scarce in the literature [5], [43]. Otherwise, we would have missed >50% of SNPs of this gene that showed association in previous studies. For the *LTA*-*TNF* and *MS4A2* genes, we only observed subtle increases of effect sizes for the SNPs that were revealed in our previous analyses, but did not evidence more SNPs reaching nominal significance (Table S3 and Figure 1). After a global FDR assessment accounting for all comparisons performed, only 10 SNPs in *MS4A2, IL4R* and *ADAM33* genes showed an FDR <5%, which were considered associated at study-wise significance (Table 2). Among these, 7 SNPs were identified to be functional, as they were either predicted to cause missense changes in the protein encoded or had empirically demonstrated regulatory roles as deduced from ENCODE project experimental data [42] (Table S4).

**Table 2:** Association summary of the 10 SNPs that resulted significantly associated with asthma after adjustments for the multiple comparisons.

Gene	rs#	Position^a^	Comparison	Allele1/ Allele2	Frecuency controls^b^	Frecuency cases^b^	OR (95% CI)^b^	*p*-value
*MS4A2*	rs569108	59863104	Asthma diagnosed ≤39	A/G	0.961	0.985	2.45 (1.39–4.33)	2.7E-04^c^
*IL4R*	rs1805015	27374180	Asthma diagnosed ≤39	T/C	0.809	0.864	1.45 (1.17–1.80)	2.1E-04^c^
*ADAM33*	rs2787093	3648462	Asthma diagnosed ≤14	T/C	0.890	0.822	0.56 (0.40–0.78)	4.9E-04
	rs628965	3649713	Asthma diagnosed ≤14	G/A	0.619	0.523	0.65 (0.50–0.84)	1.0E-04^c^
	rs628977	3649721	Asthma diagnosed ≤14	C/T	0.617	0.516	0.66 (0.51–0.85)	3.7E-04^c^
	rs630712	3650066	Asthma diagnosed ≤14	A/C	0.892	0.826	0.57 (0.41–0.80)	1.0E-04
	rs597980	3651165	Asthma diagnosed ≤14	A/G	0.440	0.326	0.60 (0.46–0.79)	1.2E-04^c^
	rs598418	3651269	Asthma diagnosed ≤14	A/G	0.618	0.523	0.65 (0.50–0.84)	1.0E-04^c^
	rs2853209	3651472	Asthma diagnosed ≤14	A/T	0.482	0.362	0.58 (0.45–0.75)	3.8E-05^c^
	rs2787095	3655943	Asthma diagnosed ≤14	C/G	0.584	0.530	1.51 (1.19–1.91)	3.8E-04^c^

tSNPs are underlined.

a According to NCBI build 36.3.

b Computed for allele 1.

c SNPs associated in previous studies.

In order to provide evidence for replication at these *loci*, we accessed the GABRIEL data, the largest GWAS meta-analysis in asthma performed in Europeans [17]. There, we were able to allocate 11 out of the 51 SNPs that reached nominal significance with asthma in our study (Table S5). Only the SNP rs1805012, located in IL4R, demonstrated in silico replication in GABRIEL (p = 5.7E-04), showing the same direction of effects as in our study.

## Discussion

In this study, we have comprehensively analyzed the association of 286 common variants of eight candidate genes with asthma and atopic asthma in a case-control Spanish sample and found associations for 10 SNPs in three of them (*MS4A2, IL4R* and *ADAM33*) after considering all tests performed. We additionally provided *in silico* replication for *IL4R* with GWAS data from the GABRIEL study.

It is well known that the age-at-onset of asthma is associated with different phenotypic characteristics [44], and it has recently evidenced that age-varying genetic associations can cause non-replication and, consequently, lead to missing important genetic associations [45]. Therefore, here we re-evaluated the association of these genes by restricting the analysis to case subjects with an age at diagnosis of asthma before a cutoff that maximized allele effects of replicated variants. This allowed us to verify that association improved for certain genes, such as *ADAM33*, as recently supported for other firm candidates [25], [46], [47], and also to gain insight in the genetic complexity of asthma associations at these candidate genes. Intriguingly, many of their effects peaked in the range of age at diagnosis between 20 and 45 years, coincidental with the age range with the maximum expression of the disease [48], [49]. It remains to be solved whether or not true biological mechanisms underlie this and previous observations [17], [25], [46]. Nevertheless, our results suggest that it will be worth considering the disease age at diagnosis in further studies, as well as in the research of improved asthma treatment and prevention.

To identify firm susceptibility genes and understand the biological processes underlying the development of the disease, replication in independent well-powered studies is essential, regardless of whether the first evidence of association was provided by a GWAS study or a candidate gene survey [50]. Besides, replication efforts allow testing the generalizability of findings in other populations, and discovering novel genetic *loci* contributing to phenotypic trait variability [51]. Particularly, testing the associations in populations of recent African ancestry will likely improve the detection of new risk variants [52], as they may offer the opportunity to refine the signal or to allocate the causal variants [53]. Our study aligns with these considerations, as it was performed in a population with sizeable North African genetic influences [28], [54], and with a sample size representing a substantially larger population of cases (>97%) than the vast majority of prior published case-control studies of these genes in unrelated individuals, although still far from optimal to detect weak effects.

Under a simplistic scenario assuming complete LD of associated SNPs with causal variants, the analyzed sample size provided a 70% power to detect a minimum risk of 1.45 for a risk allele frequency of 45% with a two-sided *p* = 0.0012 significance level for the primary outcome (asthma), and ranged from 14.6% to 52.6% for the analyses in subset of cases with atopic asthma and asthma before the age at diagnosis cutoff (Table S6). We acknowledge that risk effects of this range are in the upper bound of those expected for common variants in complex traits [55], which may have contributed to our failure to detect associations for some of the genes tested. Alternatively, our failure to find associations may possibly be attributed to: i) Our impossibility to test their association with more relevant traits or patient sub-samples (e.g. asthma drug responses [56], [57], environmental exposures [58]); ii) The use of controls self-reporting no personal or familiar history of pulmonary or allergic disease, but without a disease confirmation based on a clinical characterization (e.g. lung function measurements, SPT or specific IgE testing); iii) The lack of a true association with asthma susceptibility, as has been suggested for particularly relevant variants by meta-analyses [56]. Whichever is correct, a recently published study with on a similar sample size showed positive and negative association results fully congruent with ours [59]. In support of our results, we were able to replicate *in silico* the association of a SNP in *IL4R* in the largest GWAS study published to date that included more than 25,000 Europeans [17]. This SNP from *IL4R*, as well as few others from the same gene that were found associated in our study (rs1801275, rs1805015, and rs3024676), also demonstrated congruent effects and significant association in a recent GWAS of total IgE levels [60]. This evidence supports that, despite the enormous efforts to disentangle asthma genes such as those entailed by the GABRIEL study [17] or the EVE consortium [19], many more asthma susceptibility genes awaits its discovery.

Some recent replication studies focusing on candidate genes have utilized available arrays for genome wide genotyping [61]–[63] where common variants of many key asthma candidate genes could be insufficiently covered. In this respect, Michel et al. [59] indicated that only 37% of the previously associated SNPs from 14 candidate genes were captured by the array utilized by the same authors on the first GWAS for asthma and, surprisingly, not a single SNP from key asthma genes such as *ADAM33*, *IL4* and *CD14* was contained in their array [8]. Only after extending the study by further genotyping (and by imputation) on the same samples of their GWAS, these authors were able to consistently replicate many of the biological candidates that were missing from their GWAS [59]. We confirmed that the coverage of published GWAS for asthma performed in European populations to date has been insufficient for *ADAM33* (<30%), even in a best-case scenario using the HapMap phase 2 data as a reference for comparisons (Table S7). If array comparisons were made against the 1KGP sequencing data [38], the coverage would be even lower (Table S7). Besides, it is worth noting that the estimated coverage of these genes might be inflated, as these were implicitly derived for HapMap CEU data and the same data was used to inform the SNP contents of the array, and we have assumed that the 100% of SNPs contained in the array were successfully genotyped. Effects similar to those related to the age-of-onset of asthma, exceptionally explored [17], could have also contributed to find no association for the genes explored here in the published GWAS for asthma.

In conclusion, here we found the association of 10 common variants in three biological candidate genes (*MS4A2, IL4R* and *ADAM33*) that attained study-wise significance, and one of them was also supported by *in silico* replication in GWAS data. Therefore, we provided independent support for their role as risk factors for the amalgam of asthma phenotypes. Moreover, our results evidenced the genetic complexity at some of these susceptibility *loci* and the importance of considering age-at-onset effects. Given the low statistical power of the present study, particularly limited in the case subset analyses when considering the age at diagnosis, further studies will be needed to identify causal variants and to unravel if these genes are truly associated with asthma, with atopy or with both.

## Supporting Information

Table S1 **Relevant demographic and clinical features of GOA samples**.
(DOC)

Table S2 **Information, completion rates and Hardy-Weinberg equilibrium (HWE)** ***p*** **-values for the tSNPs.**
(DOC)

Table S3 **Association summary of SNPs with asthma, atopic asthma and asthma with age at diagnosis before the cutoff demonstrating the largest effects.**
(DOC)

Table S4 **Functional annotation of the 10 associated SNPs**.
(DOC)

Table S5 ***In silico*** **replication of the associated SNPs contained in the GABRIEL study.**
(DOC)

Table S6 **Sample sizes and statistical power for each analysis performed in a subset of cases.**
(DOC)

Table S7 **Coverage of candidate genes on commercial arrays used in asthma GWAS in samples of European ancestry.**
(DOC)

Text S1 **Supplementary methods**.
(DOC)
