# Correction: Assessing the Validity of Asthma Associations for Eight Candidate Genes and Age at Diagnosis Effects

**DOI:** 10.1371/annotation/4d0bcb4c-ade9-4393-918f-daa5e7e5f6f6

**Published:** 2013-09-13

**Authors:** María Pino-Yanes, Almudena Corrales, José Cumplido, Paloma Poza, Inmaculada Sánchez-Machín, Anselmo Sánchez-Palacios, Javier Figueroa, Orlando Acosta-Fernández, Nisa Buset, José Carlos García-Robaina, Mariano Hernández, Jesús Villar, Teresa Carrillo, Carlos Flores

There was an error in the Funding statement. The correct version of the statement is available below.

Funded by grants from the Carlos III Health Institute (http://www.isciii.es) and cofounded by the European Regional Development Fund (FEDER) "A way of making Europe": PI081383, PI1100623 and EMER07/001, and by a grant from Fundación Canaria de Investigación y Salud FUNCIS 27/07 (http://www.funcis.org) to CF. MPY was funded by a postdoctoral fellowship from Fundación Ramón Areces (http://www.fundacionareces.es). The funders had no role in study design, data collection and analysis, decision to publish, or preparation of the manuscript. 

