# Supplementary material for: Assessing the Validity of Asthma Associations for Eight Candidate Genes and Age at Diagnosis Effects
Source: PLoS One. 2013 Sep 9;8(9):e73157. doi: 10.1371/journal.pone.0073157 (PMC3767824; doi:10.1371/journal.pone.0073157)
Supplement: Table S1 — Relevant demographic and clinical features of GOA samples. (DOC) [file pone.0073157.s001.doc]

| **Table S1.** Relevant demographic and clinical features of GOA samples. | | | |
| --- | --- | --- | --- |
| Variable | Cases (n = 607) | Controls (n = 1,271) | *p*-value |
| Gender (male %) | 34.3 | 59.1 | <0.001^a^ |
| Age at recruitment, median years (P_25_-P_75_)^b^ | 34 (25-44) | 40 (32-48) | <0.001^c^ |
| Smoking habits (ever smokers, %) | 27.3 | 48.1 | <0.001^a^ |
| Clinical features: |  |  |  |
| Total IgE levels (IU/mL), median (P_25_-P_75_)^b^ | 202.0 (90.1-396.3) | NA |  |
| Age at diagnosis, median (P_25_-P_75_)^b^ | 14 (26-39) | NA |  |
| FEV1% predicted, median (P_25_-P_75_)^b^ | 83 (68-95) | NA |  |
| Allergic rhinitis (%) | 74.2 | NA |  |
| Atopic dermatitis (%) | 20.2 | NA |  |
| SPT (% positive) | 76.9 | NA |  |
| Specific IgE (% positive) | 51.6 | NA |  |
| Atopy (%) | 76.7 | NA |  |
| Asthma Severity (%): |  |  |  |
| Severe | 28.8 |  |  |
| Moderate | 23.9 |  |  |
| Mild | 47.3 |  |  |
| Treatment: |  |  |  |
| Inhaled corticoids (%) | 85.6 | NA |  |
| Oral corticoids (%) | 4.6 | NA |  |
| Long-acting β2-agonists (%) | 53.0 | NA |  |
| Short acting β2 agonists (%) | 81.8 | NA |  |
| Body mass index kg/m^2^, median (P_25_-P_75_)^b^ | 24.6 (21.1-27.5) | NA |  |
| ^a^χ^2^ test;^b^P_25_, Percentile 25; P_75_, Percentile 75; ^c^Mann-Whitney U-test; NA, not available. | | | |
