# Supplementary material for: Assessing the Validity of Asthma Associations for Eight Candidate Genes and Age at Diagnosis Effects
Source: PLoS One. 2013 Sep 9;8(9):e73157. doi: 10.1371/journal.pone.0073157 (PMC3767824; doi:10.1371/journal.pone.0073157)
Supplement: Table S2 — Information, completion rates and Hardy-Weinberg equilibrium (HWE) p -values for the tSNPs. (DOC) [file pone.0073157.s002.doc]

| **Table S2.** Information, completion rates and Hardy-Weinberg equilibrium (HWE) *p*-values for the tSNPs. | | | | | | |
| --- | --- | --- | --- | --- | --- | --- |
| Gene | rs# | Position^a^ | Effects and other designations | CR^b^ (%) | HWE Controls | HWE Cases |
| *IL13-IL4* | rs1800925 | 132020708 | -1112 C/T | 97 | 0.858 | 0.283 |
|  | rs20541 | 132023863 | Gln130Arg | 99 | **0.031** | 0.637 |
|  | rs1295683 | 132026775 |  | 96 | 0.121 | 0.607 |
|  | rs2243297 | 132027070 |  | 96 | 0.408 | 1.000 |
|  | rs2243208 | 132029050 |  | 99 | 1.000 | 0.428 |
|  | rs2243211 | 132029321 |  | 99 | 0.836 | 0.394 |
|  | rs2243302 | 132032430 |  | 97 | 0.711 | 0.704 |
|  | rs2070874 | 132037609 | Prom+33 | 91 | **0.027** | **0.026** |
|  | rs2227284 | 132040624 | SNP+3017 | 99 | 0.667 | **0.006** |
|  | rs2243281 | 132044294 |  | 97 | 0.341 | 0.789 |
| *LTA-TNF* | rs915654 | 31646476 |  | 96 | **0.010** | 0.863 |
|  | rs2844482 | 31647746 | -753 G/A | 98 | 0.602 | 1.000 |
|  | rs2071590 | 31647747 |  | 97 | **0.045** | 0.852 |
|  | rs2239704 | 31648120 |  | 96 | 0.518 | 0.793 |
|  | rs909253 | 31648292 | NcoI Intron 1, 252 A/G | 98 | 0.721 | 0.684 |
|  | rs746868 | 31648408 |  | 99 | 0.643 | 0.664 |
|  | rs2229092 | 31648736 |  | 99 | 0.826 | 1.000 |
|  | rs1041981 | 31648763 |  | 94 | 0.609 | **0.026** |
|  | rs1799724 | 31650461 | -857 C/T | 99 | 0.672 | 0.117 |
|  | rs1800629 | 31651010 | -308 G/A | 98 | 0.612 | 0.185 |
|  | rs1800610 | 31651806 |  | 99 | 1.000 | 0.056 |
| *CD14* | rs2915863 | 139994561 | -1601 intronic C/T | 92 | **0.002** | 0.223 |
|  | rs2569191 | 139994087 |  | 97 | 0.059 | 0.621 |
|  | rs2569190 | 139993100 | -159 C/T | 98 | 0.231 | 1.000 |
|  | rs4914 | 139991652 | 1309 synonymous coding C/G | 98 | 0.214 | 0.682 |
|  | rs2563298 | 139991499 | 1462 C/A | 93 | 0.497 | 0.741 |
| *MS4A2* | rs12361312 | 59609172 |  | 98 | **9.9E-06** | 0.358 |
|  | rs513986 | 59609429 |  | 99 | 0.728 | 0.799 |
|  | rs2583477 | 59610431 |  | 97 | 0.605 | 0.679 |
|  | rs573790 | 59611961 |  | 98 | 0.249 | 0.653 |
|  | rs1441586 | 59612604 | -109 C/T | 98 | 0.425 | 0.680 |
|  | rs569108 | 59619680 | Gly237Glu | 99 | 0.433 | **0.042** |
|  | rs2847655 | 59622247 | Rsalex7 | 99 | 0.272 | 0.616 |
| *ADRB2* | rs12654778 | 148185934 | -654 A/G | 98 | 0.051 | 0.547 |
|  | rs11959427 | 148186221 |  | 94 | 0.285 | 0.857 |
|  | rs1042713 | 148186633 | Arg16Gly | 98 | 0.129 | 0.800 |
|  | rs1042718 | 148187110 |  | 97 | 0.126 | **0.030** |
|  | rs1042719 | 148187640 |  | 97 | 0.319 | 0.314 |
|  | rs1042720 | 148187826 |  | 98 | 1.000 | 0.785 |
|  | rs4705271 | 148192184 |  | 99 | 0.091 | 0.379 |
| *ADAM33* | rs554743 | 3610142 |  | 98 | 0.884 | 0.102 |
|  | rs2853213 | 3609840 |  | 98 | 0.660 | 0.195 |
|  | rs17548962 | 3609803 |  | 98 | 1.000 | 1.000 |
|  | rs570269^c^ | 3607647 |  | 98 | 0.109 | 0.793 |
|  | rs2853210 | 3606211 |  | 97 | 0.815 | 0.910 |
|  | rs597165 | 3605804 |  | 98 | 1.000 | 0.602 |
|  | rs2787095 | 3603943 |  | 99 | 0.449 | 0.068 |
|  | rs511898 | 3603085 | 216_F_+1 Intron | 99 | 0.622 | 0.663 |
|  | rs528557 | 3599742 | 216_S_2 ORF (Gly717Gly ) | 97 | 0.883 | 0.262 |
|  | rs574174 | 3598694 | 216_ST_+7 Intron | 98 | 0.924 | 0.433 |
|  | rs2280091 | 3598234 | 216_T_1 ORF (Thr764Met) | 98 | 0.320 | 0.314 |
|  | rs2280090 | 3598205 | 216_T_2 ORF (Ser774Pro) | 99 | 0.152 | 0.254 |
|  | rs678881 | 3597803 |  | 98 | 1.000 | 0.917 |
|  | rs628977 | 3597721 |  | 98 | 0.433 | 1.000 |
|  | rs512625 | 3596378 |  | 98 | 0.623 | 0.848 |
| *IL4R* | rs2057768 | 27229596 | -3223 C/T | 98 | 0.423 | 1.000 |
|  | rs1110470 | 27243928 |  | 98 | 0.954 | 0.115 |
|  | rs2283563 | 27253855 |  | 98 | 0.519 | 0.693 |
|  | rs3024537 | 27260320 |  | 99 | 0.382 | 0.626 |
|  | rs1805010 | 27263704 | Ile50Val | 97 | 0.730 | 0.184 |
|  | rs2301807 | 27265599 |  | 97 | 0.698 | 1.000 |
|  | rs3024585 | 27267345 |  | 98 | 0.122 | 0.624 |
|  | rs3024586 | 27267542 |  | 99 | **0.027** | 0.572 |
|  | rs2283561 | 27268668 |  | 95 | 0.859 | 0.800 |
|  | rs3024607 | 27271112 |  | 99 | 0.359 | 0.759 |
|  | rs3024613 | 27271754 |  | 98 | 0.955 | 0.461 |
|  | rs3024632 | 27273797 |  | 99 | 0.563 | 0.239 |
|  | rs3024676 | 27281059 |  | 99 | 0.165 | 0.175 |
|  | rs2234897 | 27281113 |  | 100 | 1.000 | 0.224 |
|  | rs1805011 | 27281373 | Glu375Ala | 99 | 0.185 | 0.866 |
|  | rs1805012 | 27281465 | Cys406Arg | 99 | 0.156 | 0.173 |
|  | rs1805013 | 27281481 | Ser411Leu | 99 | 0.140 | 1.000 |
|  | rs1805015 | 27281681 | Ser478Pro | 98 | 0.359 | **0.039** |
|  | rs1801275 | 27281901 | Gln551Arg | 98 | **0.026** | 0.104 |
|  | rs1805016 | 27282428 | Ser727Ala | 98 | 0.457 | 0.715 |
|  | rs1049631 | 27283043 |  | 98 | **0.040** | **0.032** |
| ^a^According to NCBI build 36.3;^b^Completion rate; ^c^No distinction was made between C and the new T allele for this analysis. Nominal significant deviations in bold. | | | | | | |
