# Supplementary material for: Assessing the Validity of Asthma Associations for Eight Candidate Genes and Age at Diagnosis Effects
Source: PLoS One. 2013 Sep 9;8(9):e73157. doi: 10.1371/journal.pone.0073157 (PMC3767824; doi:10.1371/journal.pone.0073157)
Supplement: Table S3 — Association summary of SNPs with asthma, atopic asthma and asthma with age at diagnosis before the cutoff demonstrating the largest effects. (DOC) [file pone.0073157.s003.doc]

| **Table S3.** Association summary of SNPs with asthma, atopic asthma and asthma with age-at-diagnosis of the disease before the cutoff demonstrating the largest effects at replicated SNPs. | | | | | | | | | | | | | | | | |
| --- | --- | --- | --- | --- | --- | --- | --- | --- | --- | --- | --- | --- | --- | --- | --- | --- |
|  |  |  |  |  | Frequency allele 1 | | | |  | OR ± SE^c^ | | |  | *p*-value | | |
| Gene | rs# | Position^a^ | Allele1/Allele2 | Rsq^b^ | Controls | Asthma | Atopic asthma | Asthma before age-at-diagnosis cutoff |  | Asthma | Atopic asthma | Asthma before age-at-diagnosis cutoff |  | Asthma | Atopic asthma | Asthma before age-at-diagnosis cutoff |
| *IL13-IL4* | rs115008099 | 131991881 | C/T | 0.79 | 0.823 | 0.805 | 0.792 | NA |  | 0.84 ± 0.10 | 0.78 ± 0.12 | NA |  | 0.094 | **0.035** | NA |
|  | rs1881457 | 131992409 | A/C | 0.80 | 0.820 | 0.801 | 0.789 | NA |  | 0.84 ± 0.10 | 0.78 ± 0.12 | NA |  | 0.092 | **0.035**^d^ | NA |
|  | rs1800925 | 131992809 | C/T |  | 0.801 | 0.779 | 0.764 | NA |  | 0.85 ± 0.10 | 0.78 ± 0.11 | NA |  | 0.100 | **0.037**^d^ | NA |
|  | rs1295687 | 131994462 | G/C | 0.37 | 0.950 | 0.955 | 0.948 | NA |  | 1.32 ± 0.28 | 0.89 ± 0.31 | NA |  | 0.319 | 0.697 | NA |
|  | rs1295686 | 131995843 | C/T | 0.96 | 0.848 | 0.843 | 0.822 | NA |  | 0.94 ± 0.10 | 0.82 ± 0.11 | NA |  | 0.513 | 0.080 | NA |
|  | rs20541 | 131995964 | G/A |  | 0.854 | 0.848 | 0.826 | NA |  | 0.94 ± 0.10 | 0.83 ± 0.11 | NA |  | 0.462 | 0.066 | NA |
|  | rs1295685 | 131996445 | G/A | 0.96 | 0.846 | 0.841 | 0.818 | NA |  | 0.94 ± 0.10 | 0.82 ± 0.11 | NA |  | 0.505 | 0.069 | NA |
|  | rs848 | 131996500 | C/A | 0.96 | 0.846 | 0.840 | 0.818 | NA |  | 0.94 ± 0.10 | 0.82 ± 0.11 | NA |  | 0.506 | 0.069 | NA |
|  | rs847 | 131996669 | C/T | 0.96 | 0.845 | 0.840 | 0.818 | NA |  | 0.94 ± 0.10 | 0.82 ± 0.11 | NA |  | 0.509 | 0.069 | NA |
|  | rs2069757 | 131998413 | G/A | 0.97 | 0.927 | 0.926 | 0.913 | NA |  | 0.96 ± 0.14 | 0.82 ± 0.16 | NA |  | 0.767 | 0.197 | NA |
|  | rs1295683 | 131998876 | G/A |  | 0.919 | 0.912 | 0.899 | NA |  | 0.93 ± 0.14 | 0.83 ± 0.15 | NA |  | 0.400 | 0.109 | NA |
|  | rs2243297 | 131999171 | T/A |  | 0.963 | 0.966 | 0.956 | NA |  | 1.01 ± 0.18 | 0.80 ± 0.20 | NA |  | 0.733 | 0.398 | NA |
|  | rs2243204 | 131999494 | C/T | 0.96 | 0.926 | 0.925 | 0.912 | NA |  | 0.96 ± 0.14 | 0.82 ± 0.16 | NA |  | 0.786 | 0.196 | NA |
|  | rs2243208 | 132001151 | A/G |  | 0.923 | 0.914 | 0.902 | NA |  | 0.91 ± 0.13 | 0.79 ± 0.15 | NA |  | 0.367 | 0.082 | NA |
|  | rs2243210 | 132001386 | G/A | 0.97 | 0.925 | 0.920 | 0.911 | NA |  | 0.92 ± 0.13 | 0.83 ± 0.15 | NA |  | 0.553 | 0.226 | NA |
|  | rs2243211 | 132001422 | C/A |  | 0.926 | 0.922 | 0.914 | NA |  | 0.93 ± 0.13 | 0.83 ± 0.15 | NA |  | 0.572 | 0.263 | NA |
|  | rs2243217 | 132001933 | A/T | 0.91 | 0.922 | 0.916 | 0.906 | NA |  | 0.90 ± 0.13 | 0.80 ± 0.15 | NA |  | 0.449 | 0.147 | NA |
|  | rs2243218 | 132002024 | G/A | 0.89 | 0.922 | 0.916 | 0.907 | NA |  | 0.91 ± 0.14 | 0.80 ± 0.16 | NA |  | 0.470 | 0.155 | NA |
|  | rs2243219 | 132002125 | A/G | 0.88 | 0.921 | 0.915 | 0.905 | NA |  | 0.91 ± 0.14 | 0.80 ± 0.16 | NA |  | 0.472 | 0.157 | NA |
|  | rs2243221 | 132002844 | C/T | 0.87 | 0.923 | 0.918 | 0.908 | NA |  | 0.90 ± 0.14 | 0.80 ± 0.16 | NA |  | 0.459 | 0.156 | NA |
|  | rs2243300 | 132004086 | G/T | 0.81 | 0.922 | 0.917 | 0.908 | NA |  | 0.90 ± 0.14 | 0.80 ± 0.16 | NA |  | 0.469 | 0.166 | NA |
|  | rs2243228 | 132004363 | A/C | 0.75 | 0.915 | 0.911 | 0.902 | NA |  | 0.92 ± 0.14 | 0.81 ± 0.17 | NA |  | 0.562 | 0.209 | NA |
|  | rs2243302 | 132004531 | G/A |  | 0.866 | 0.876 | 0.870 | NA |  | 1.09 ± 0.12 | 1.04 ± 0.14 | NA |  | 0.460 | 0.874 | NA |
|  | rs762534 | 132004756 | C/A | 0.66 | 0.913 | 0.910 | 0.901 | NA |  | 0.92 ± 0.15 | 0.80 ± 0.18 | NA |  | 0.589 | 0.209 | NA |
|  | rs2243248 | 132008644 | T/G | 0.59 | 0.929 | 0.933 | 0.932 | NA |  | 1.09 ± 0.18 | 1.08 ± 0.22 | NA |  | 0.627 | 0.719 | NA |
|  | rs2243250 | 132009154 | C/T | 0.97 | 0.853 | 0.855 | 0.856 | NA |  | 1.00 ± 0.10 | 1.02 ± 0.12 | NA |  | 0.990 | 0.877 | NA |
|  | rs2070874 | 132009710 | C/T |  | 0.868 | 0.867 | 0.867 | NA |  | 1.00 ± 0.10 | 1.02 ± 0.12 | NA |  | 0.816 | 0.883 | NA |
|  | rs734244 | 132010726 | C/T | 0.96 | 0.853 | 0.854 | 0.856 | NA |  | 1.00 ± 0.10 | 1.02 ± 0.12 | NA |  | 0.987 | 0.867 | NA |
|  | rs2227284 | 132012725 | G/T |  | 0.728 | 0.734 | 0.727 | NA |  | 1.04 ± 0.08 | 1.02 ± 0.10 | NA |  | 0.771 | 0.932 | NA |
|  | rs2227282 | 132013179 | G/C | 0.95 | 0.739 | 0.749 | 0.744 | NA |  | 1.04 ± 0.08 | 1.02 ± 0.10 | NA |  | 0.625 | 0.821 | NA |
|  | rs2243263 | 132013299 | G/C | 0.97 | 0.898 | 0.911 | 0.907 | NA |  | 1.15 ± 0.12 | 1.10 ± 0.15 | NA |  | 0.247 | 0.504 | NA |
|  | rs2243266 | 132013789 | G/A | 0.94 | 0.851 | 0.852 | 0.853 | NA |  | 0.99 ± 0.10 | 1.01 ± 0.12 | NA |  | 0.927 | 0.952 | NA |
|  | rs2243267 | 132013886 | G/C | 0.94 | 0.851 | 0.852 | 0.852 | NA |  | 0.99 ± 0.10 | 1.01 ± 0.12 | NA |  | 0.927 | 0.953 | NA |
|  | rs2243268 | 132013963 | A/C | 0.94 | 0.851 | 0.852 | 0.852 | NA |  | 0.99 ± 0.10 | 1.01 ± 0.12 | NA |  | 0.926 | 0.954 | NA |
|  | rs2243270 | 132014109 | A/G | 0.92 | 0.841 | 0.838 | 0.837 | NA |  | 0.97 ± 0.10 | 0.97 ± 0.12 | NA |  | 0.724 | 0.779 | NA |
|  | rs2243274 | 132014832 | G/A | 0.91 | 0.841 | 0.838 | 0.837 | NA |  | 0.97 ± 0.10 | 0.97 ± 0.12 | NA |  | 0.723 | 0.778 | NA |
|  | rs2243281 | 132016395 | T/C |  | 0.901 | 0.915 | 0.910 | NA |  | 1.15 ± 0.12 | 1.10 ± 0.15 | NA |  | 0.218 | 0.514 | NA |
|  | rs2243282 | 132016554 | C/A | 0.93 | 0.851 | 0.851 | 0.852 | NA |  | 0.99 ± 0.10 | 1.01 ± 0.12 | NA |  | 0.922 | 0.959 | NA |
|  | rs2243284 | 132016992 | G/A | 0.89 | 0.842 | 0.838 | 0.838 | NA |  | 0.97 ± 0.10 | 0.97 ± 0.12 | NA |  | 0.744 | 0.785 | NA |
|  | rs2243285 | 132016993 | G/T | 0.97 | 0.900 | 0.913 | 0.909 | NA |  | 1.16 ± 0.12 | 1.11 ± 0.15 | NA |  | 0.241 | 0.487 | NA |
|  | rs2243288 | 132017944 | A/G | 0.89 | 0.840 | 0.836 | 0.836 | NA |  | 0.97 ± 0.10 | 0.97 ± 0.12 | NA |  | 0.717 | 0.767 | NA |
|  | rs2243289 | 132018132 | A/G | 0.91 | 0.850 | 0.850 | 0.851 | NA |  | 0.99 ± 0.10 | 1.01 ± 0.12 | NA |  | 0.918 | 0.968 | NA |
|  | rs2243290 | 132018169 | C/A | 0.91 | 0.850 | 0.850 | 0.851 | NA |  | 0.99 ± 0.10 | 1.01 ± 0.12 | NA |  | 0.918 | 0.968 | NA |
|  | rs2243291 | 132018983 | G/C | 0.89 | 0.840 | 0.836 | 0.836 | NA |  | 0.97 ± 0.10 | 0.97 ± 0.12 | NA |  | 0.717 | 0.767 | NA |
|  | rs2243293 | 132019799 | A/G | 0.89 | 0.747 | 0.760 | 0.757 | NA |  | 1.06 ± 0.09 | 1.06 ± 0.10 | NA |  | 0.467 | 0.600 | NA |
| *CD14* | rs2569188 | 140008419 | A/G | 0.98 | 0.483 | 0.503 | 0.485 | NA |  | 1.07 ± 0.07 | 1.00 ± 0.09 | NA |  | 0.363 | 0.992 | NA |
|  | rs58150262 | 140008476 | C/T | 0.87 | 0.789 | 0.773 | 0.768 | NA |  | 0.92 ± 0.09 | 0.88 ± 0.11 | NA |  | 0.358 | 0.235 | NA |
|  | rs2563315 | 140008505 | G/A | 0.90 | 0.271 | 0.276 | 0.253 | NA |  | 1.02 ± 0.08 | 0.90 ± 0.10 | NA |  | 0.808 | 0.285 | NA |
|  | rs7723769 | 140008880 | C/T | 0.87 | 0.789 | 0.773 | 0.768 | NA |  | 0.92 ± 0.09 | 0.88 ± 0.11 | NA |  | 0.358 | 0.235 | NA |
|  | rs56746017 | 140008901 | T/A | 0.87 | 0.789 | 0.773 | 0.768 | NA |  | 0.92 ± 0.09 | 0.88 ± 0.11 | NA |  | 0.358 | 0.235 | NA |
|  | rs7728187 | 140009273 | C/G | 0.86 | 0.790 | 0.774 | 0.769 | NA |  | 0.92 ± 0.09 | 0.88 ± 0.11 | NA |  | 0.358 | 0.235 | NA |
|  | rs2563298 | 140011315 | A/C |  | 0.258 | 0.263 | 0.239 | NA |  | 1.02 ± 0.08 | 0.89 ± 0.10 | NA |  | 0.774 | 0.322 | NA |
|  | rs4914 | 140011468 | G/C |  | 0.116 | 0.113 | 0.105 | NA |  | 0.96 ± 0.11 | 0.90 ± 0.14 | NA |  | 0.670 | 0.411 | NA |
|  | rs2569190 | 140012916 | G/A |  | 0.482 | 0.494 | 0.487 | NA |  | 1.06 ± 0.07 | 1.00 ± 0.08 | NA |  | 0.276 | 0.883 | NA |
|  | rs5744455 | 140013307 | G/A | 0.87 | 0.786 | 0.771 | 0.765 | NA |  | 0.92 ± 0.09 | 0.88 ± 0.11 | NA |  | 0.357 | 0.232 | NA |
|  | rs5744454 | 140013567 | T/G | 0.93 | 0.778 | 0.762 | 0.756 | NA |  | 0.92 ± 0.09 | 0.88 ± 0.10 | NA |  | 0.356 | 0.233 | NA |
|  | rs2569191 | 140013903 | T/C |  | 0.484 | 0.496 | 0.488 | NA |  | 1.06 ± 0.07 | 1.00 ± 0.08 | NA |  | 0.355 | 0.890 | NA |
|  | rs3138078 | 140014117 | C/A | 0.91 | 0.782 | 0.767 | 0.761 | NA |  | 0.92 ± 0.09 | 0.88 ± 0.11 | NA |  | 0.372 | 0.239 | NA |
|  | rs2915863 | 140014377 | T/C |  | 0.460 | 0.444 | 0.460 | NA |  | 1.04 ± 0.07 | 0.99 ± 0.08 | NA |  | 0.571 | 0.918 | NA |
|  | rs3138076 | 140014613 | T/C | 0.94 | 0.775 | 0.759 | 0.753 | NA |  | 0.93 ± 0.09 | 0.89 ± 0.10 | NA |  | 0.385 | 0.246 | NA |
|  | rs2569192 | 140015208 | C/G | 0.95 | 0.258 | 0.260 | 0.237 | NA |  | 1.01 ± 0.08 | 0.89 ± 0.10 | NA |  | 0.920 | 0.242 | NA |
|  | rs2569193 | 140015495 | A/G | 0.95 | 0.258 | 0.261 | 0.237 | NA |  | 1.01 ± 0.08 | 0.89 ± 0.10 | NA |  | 0.920 | 0.242 | NA |
|  | rs2563310 | 140015741 | G/A | 0.98 | 0.484 | 0.502 | 0.484 | NA |  | 1.06 ± 0.07 | 1.00 ± 0.09 | NA |  | 0.429 | 0.962 | NA |
|  | rs3138074 | 140015932 | A/T | 0.93 | 0.775 | 0.759 | 0.753 | NA |  | 0.93 ± 0.09 | 0.89 ± 0.10 | NA |  | 0.385 | 0.245 | NA |
|  | rs5744441 | 140016847 | G/A | 0.90 | 0.780 | 0.765 | 0.759 | NA |  | 0.93 ± 0.09 | 0.89 ± 0.11 | NA |  | 0.384 | 0.245 | NA |
|  | rs4912717 | 140018235 | A/G | 0.93 | 0.260 | 0.262 | 0.239 | NA |  | 1.01 ± 0.08 | 0.89 ± 0.10 | NA |  | 0.923 | 0.241 | NA |
|  | rs4912718 | 140018378 | T/C | 0.97 | 0.485 | 0.503 | 0.486 | NA |  | 1.06 ± 0.07 | 1.00 ± 0.09 | NA |  | 0.428 | 0.963 | NA |
| *ADRB2* | rs11746634 | 148202668 | G/C | 0.95 | 0.628 | 0.618 | 0.610 | NA |  | 0.96 ± 0.08 | 0.92 ± 0.09 | NA |  | 0.586 | 0.366 | NA |
|  | rs11168067 | 148202801 | G/A | 0.95 | 0.628 | 0.618 | 0.610 | NA |  | 0.96 ± 0.08 | 0.92 ± 0.09 | NA |  | 0.586 | 0.365 | NA |
|  | rs9325122 | 148202936 | T/C | 0.92 | 0.649 | 0.641 | 0.631 | NA |  | 0.96 ± 0.08 | 0.92 ± 0.09 | NA |  | 0.611 | 0.344 | NA |
|  | rs11957351 | 148203104 | T/C | 0.37 | 0.370 | 0.355 | 0.353 | NA |  | 0.84 ± 0.12 | 0.82 ± 0.14 | NA |  | 0.151 | 0.177 | NA |
|  | rs11960649 | 148203144 | C/A | 0.87 | 0.605 | 0.594 | 0.587 | NA |  | 0.95 ± 0.08 | 0.92 ± 0.09 | NA |  | 0.543 | 0.357 | NA |
|  | rs35684381 | 148203236 | T/C | 0.73 | 0.818 | 0.823 | 0.814 | NA |  | 1.04 ± 0.11 | 0.96 ± 0.13 | NA |  | 0.720 | 0.768 | NA |
|  | rs1432622 | 148203762 | C/T | 0.96 | 0.628 | 0.618 | 0.610 | NA |  | 0.96 ± 0.07 | 0.92 ± 0.09 | NA |  | 0.586 | 0.366 | NA |
|  | rs1432623 | 148204008 | T/C | 0.96 | 0.628 | 0.618 | 0.610 | NA |  | 0.96 ± 0.07 | 0.92 ± 0.09 | NA |  | 0.586 | 0.365 | NA |
|  | rs11168068 | 148204121 | T/C | 0.96 | 0.628 | 0.618 | 0.610 | NA |  | 0.96 ± 0.07 | 0.92 ± 0.09 | NA |  | 0.586 | 0.365 | NA |
|  | rs17778257 | 148204577 | T/A | 0.98 | 0.402 | 0.395 | 0.377 | NA |  | 0.98 ± 0.07 | 0.91 ± 0.09 | NA |  | 0.726 | 0.248 | NA |
|  | rs2400706 | 148204864 | C/T | 0.96 | 0.773 | 0.777 | 0.768 | NA |  | 1.02 ± 0.09 | 0.97 ± 0.10 | NA |  | 0.835 | 0.732 | NA |
|  | rs2895795 | 148204966 | T/A | 0.96 | 0.773 | 0.777 | 0.768 | NA |  | 1.02 ± 0.09 | 0.97 ± 0.10 | NA |  | 0.836 | 0.732 | NA |
|  | rs2400707 | 148205052 | G/A | 0.97 | 0.628 | 0.618 | 0.610 | NA |  | 0.96 ± 0.07 | 0.92 ± 0.09 | NA |  | 0.586 | 0.365 | NA |
|  | rs2053044 | 148205372 | G/A | 0.97 | 0.628 | 0.618 | 0.610 | NA |  | 0.96 ± 0.07 | 0.92 ± 0.09 | NA |  | 0.586 | 0.365 | NA |
|  | rs12654778 | 148205741 | A/G |  | 0.399 | 0.388 | 0.371 | NA |  | 0.96 ± 0.07 | 0.89 ± 0.09 | NA |  | 0.565 | 0.184 | NA |
|  | rs11168070 | 148205927 | C/G | 0.97 | 0.641 | 0.633 | 0.623 | NA |  | 0.96 ± 0.07 | 0.92 ± 0.09 | NA |  | 0.617 | 0.349 | NA |
|  | rs11959427 | 148206028 | T/C |  | 0.639 | 0.641 | 0.633 | NA |  | 0.97 ± 0.07 | 0.93 ± 0.09 | NA |  | 0.979 | 0.706 | NA |
|  | rs1042711 | 148206348 | T/C | 0.97 | 0.642 | 0.635 | 0.624 | NA |  | 0.97 ± 0.07 | 0.92 ± 0.09 | NA |  | 0.673 | 0.374 | NA |
|  | rs1801704 | 148206375 | T/C | 0.98 | 0.642 | 0.636 | 0.626 | NA |  | 0.97 ± 0.07 | 0.93 ± 0.09 | NA |  | 0.691 | 0.409 | NA |
|  | rs1042713 | 148206440 | A/G |  | 0.416 | 0.414 | 0.393 | NA |  | 0.99 ± 0.07 | 0.92 ± 0.09 | NA |  | 0.872 | 0.250 | NA |
|  | rs1042714 | 148206473 | C/G | 0.96 | 0.644 | 0.635 | 0.626 | NA |  | 0.96 ± 0.08 | 0.92 ± 0.09 | NA |  | 0.600 | 0.371 | NA |
|  | rs1042717 | 148206646 | G/A | 0.97 | 0.773 | 0.779 | 0.771 | NA |  | 1.03 ± 0.09 | 0.99 ± 0.10 | NA |  | 0.721 | 0.891 | NA |
|  | rs1042718 | 148206917 | C/A |  | 0.816 | 0.813 | 0.807 | NA |  | 0.97 ± 0.09 | 0.95 ± 0.11 | NA |  | 0.703 | 0.552 | NA |
|  | rs1042719 | 148207447 | G/C |  | 0.687 | 0.716 | 0.709 | NA |  | 1.16 ± 0.08 | 1.13 ± 0.09 | NA |  | 0.086 | 0.265 | NA |
|  | rs1042720 | 148207633 | G/A |  | 0.638 | 0.653 | 0.647 | NA |  | 1.10 ± 0.08 | 1.08 ± 0.09 | NA |  | 0.418 | 0.657 | NA |
|  | rs7702861 | 148211858 | C/G | 0.55 | 0.776 | 0.779 | 0.777 | NA |  | 1.04 ± 0.11 | 1.02 ± 0.14 | NA |  | 0.721 | 0.896 | NA |
|  | rs4705271 | 148211991 | C/A |  | 0.801 | 0.797 | 0.792 | NA |  | 0.97 ± 0.09 | 0.94 ± 0.10 | NA |  | 0.793 | 0.636 | NA |
| *LTA-TNF* | rs2009658 | 31538244 | C/G | 0.96 | 0.838 | 0.805 | 0.802 | 0.795 |  | 0.80 ± 0.09 | 0.78 ± 0.11 | 0.74 ± 0.12 |  | **0.015** | **0.022** | **0.013** |
|  | rs915654 | 31538497 | A/T |  | 0.350 | 0.391 | 0.387 | 0.387 |  | 1.19 ± 0.07 | 1.16 ± 0.09 | 1.17 ± 0.10 |  | **0.025** | 0.085 | 0.132 |
|  | rs2844482 | 31539767 | C/T |  | 0.837 | 0.804 | 0.802 | 0.794 |  | 0.80 ± 0.09 | 0.78 ± 0.11 | 0.74 ± 0.12 |  | **0.020** ^d^ | **0.033** ^d^ | **0.018** ^d^ |
|  | rs2071590 | 31539768 | G/A |  | 0.622 | 0.673 | 0.676 | 0.699 |  | 1.20 ± 0.07 | 1.25 ± 0.09 | 1.31 ± 0.10 |  | **0.013** ^d^ | **0.013** ^d^ | **0.003** ^d^ |
|  | rs1800683 | 31540071 | A/G | 0.99 | 0.276 | 0.284 | 0.274 | 0.273 |  | 1.02 ± 0.08 | 0.98 ± 0.10 | 0.95 ± 0.10 |  | 0.854 | 0.829 | 0.642 |
|  | rs2239704 | 31540141 | C/A |  | 0.575 | 0.615 | 0.624 | 0.625 |  | 1.17 ± 0.07 | 1.23 ± 0.09 | 1.24 ± 0.10 |  | 0.055 | **0.029** | 0.071 |
|  | rs909253 | 31540313 | G/A |  | 0.274 | 0.279 | 0.268 | 0.270 |  | 1.02 ± 0.08 | 0.98 ± 0.10 | 0.96 ± 0.10 |  | 0.978 | 0.694 | 0.651 |
|  | rs746868 | 31540429 | G/C |  | 0.578 | 0.621 | 0.627 | 0.633 |  | 1.17 ± 0.07 | 1.23 ± 0.09 | 1.24 ± 0.10 |  | **0.037** | **0.025** | **0.038** |
|  | rs2229094 | 31540556 | T/C | 0.99 | 0.699 | 0.662 | 0.645 | 0.639 |  | 0.85 ± 0.08 | 0.79 ± 0.09 | 0.77 ± 0.10 |  | **0.035** | **0.007** | **0.007** |
|  | rs2229092 | 31540757 | A/C |  | 0.932 | 0.940 | 0.942 | 0.943 |  | 1.15 ± 0.07 | 1.18± 0.09 | 1.16 ± 0.10 |  | 0.360 | 0.354 | 0.402 |
|  | rs1041981 | 31540784 | A/C |  | 0.270 | 0.278 | 0.266 | 0.265 |  | 1.02 ± 0.08 | 0.98 ± 0.10 | 0.96 ± 0.10 |  | 0.841 | 0.774 | 0.660 |
|  | rs1799964 | 31542308 | T/C | 0.84 | 0.786 | 0.753 | 0.744 | 0.733 |  | 0.81 ± 0.09 | 0.76 ± 0.11 | 0.71 ± 0.12 |  | **0.024** ^d^ | **0.011** ^d^ | **0.004** ^d^ |
|  | rs1800630 | 31542476 | C/A | 0.98 | 0.838 | 0.803 | 0.799 | 0.792 |  | 0.79 ± 0.09 | 0.77 ± 0.11 | 0.74 ± 0.12 |  | **0.012** ^d^ | **0.016** ^d^ | **0.010** ^d^ |
|  | rs1799724 | 31542482 | C/T |  | 0.888 | 0.912 | 0.921 | 0.913 |  | 1.30 ± 0.12 | 1.42 ± 0.15 | 1.27 ± 0.16 |  | **0.021** ^d^ | **0.009** ^d^ | 0.068 |
|  | rs1800629 | 31543031 | G/A |  | 0.870 | 0.855 | 0.846 | 0.861 |  | 0.91 ± 0.10 | 0.83 ± 0.12 | 0.96 ± 0.14 |  | 0.321 | 0.123 | 0.797 |
|  | rs1800610 | 31543827 | G/A |  | 0.889 | 0.916 | 0.920 | 0.911 |  | 1.31 ± 0.12 | 1.45 ± 0.15 | 1.29 ± 0.16 |  | **0.014** | **0.016** | 0.127 |
| *MS4A2* | rs513986 | 59852853 | C/T |  | 0.570 | 0.595 | 0.597 | 0.589 |  | 1.11 ± 0.07 | 1.11 ± 0.09 | 1.07 ± 0.08 |  | 0.138 | 0.194 | 0.347 |
|  | rs1286165 | 59853010 | A/G | 0.99 | 0.570 | 0.593 | 0.594 | 0.585 |  | 1.11 ± 0.07 | 1.11 ± 0.09 | 1.07 ± 0.08 |  | 0.169 | 0.233 | 0.420 |
|  | rs2847659 | 59853852 | A/G | 0.99 | 0.570 | 0.593 | 0.594 | 0.585 |  | 1.11 ± 0.07 | 1.11 ± 0.09 | 1.07 ± 0.08 |  | 0.169 | 0.233 | 0.420 |
|  | rs2583477 | 59853855 | T/C |  | 0.519 | 0.553 | 0.553 | 0.550 |  | 1.15 ± 0.07 | 1.15 ± 0.09 | 1.14 ± 0.08 |  | **0.038** | 0.102 | 0.097 |
|  | rs10792255 | 59854313 | T/C | 0.81 | 0.233 | 0.251 | 0.247 | 0.250 |  | 1.15 ± 0.09 | 1.10 ± 0.11 | 1.14 ± 0.10 |  | 0.136 | 0.372 | 0.222 |
|  | rs524748 | 59854572 | C/T | 0.99 | 0.571 | 0.594 | 0.596 | 0.586 |  | 1.11 ± 0.07 | 1.11 ± 0.09 | 1.07 ± 0.08 |  | 0.169 | 0.232 | 0.420 |
|  | rs573790 | 59855385 | T/C |  | 0.333 | 0.351 | 0.342 | 0.349 |  | 1.09 ± 0.07 | 1.05 ± 0.09 | 1.08 ± 0.08 |  | 0.306 | 0.648 | 0.449 |
|  | rs1441586 | 59856028 | T/C |  | 0.534 | 0.543 | 0.527 | 0.551 |  | 1.06 ± 0.07 | 1.00 ± 0.09 | 1.10 ± 0.08 |  | 0.636 | 0.722 | 0.398 |
|  | rs2583476 | 59857581 | G/A | 0.98 | 0.561 | 0.561 | 0.549 | 0.563 |  | 1.00 ± 0.07 | 0.95 ± 0.09 | 1.00 ± 0.08 |  | 0.943 | 0.547 | 0.958 |
|  | rs2847663 | 59858036 | C/G | 0.98 | 0.561 | 0.561 | 0.549 | 0.563 |  | 1.00 ± 0.07 | 0.95 ± 0.09 | 1.01 ± 0.08 |  | 0.946 | 0.550 | 0.954 |
|  | rs2847664 | 59858497 | G/A | 0.98 | 0.561 | 0.561 | 0.549 | 0.563 |  | 1.00 ± 0.07 | 0.95 ± 0.09 | 1.01 ± 0.08 |  | 0.955 | 0.558 | 0.945 |
|  | rs556917 | 59858712 | A/T | 0.98 | 0.522 | 0.536 | 0.527 | 0.547 |  | 1.06 ± 0.07 | 1.02 ± 0.09 | 1.10 ± 0.08 |  | 0.422 | 0.839 | 0.221 |
|  | rs2847666 | 59859576 | A/G | 0.98 | 0.567 | 0.572 | 0.558 | 0.577 |  | 1.02 ± 0.07 | 0.96 ± 0.09 | 1.04 ± 0.08 |  | 0.829 | 0.636 | 0.665 |
|  | rs2847667 | 59859609 | C/T | 0.98 | 0.567 | 0.572 | 0.558 | 0.577 |  | 1.02 ± 0.07 | 0.96 ± 0.09 | 1.04 ± 0.08 |  | 0.826 | 0.639 | 0.662 |
|  | rs502581 | 59860178 | G/T | 0.98 | 0.521 | 0.536 | 0.528 | 0.547 |  | 1.06 ± 0.07 | 1.02 ± 0.09 | 1.11 ± 0.08 |  | 0.390 | 0.780 | 0.200 |
|  | rs2583471 | 59861814 | G/A | 0.98 | 0.560 | 0.561 | 0.550 | 0.564 |  | 1.00 ± 0.07 | 0.96 ± 0.09 | 1.01 ± 0.08 |  | 0.998 | 0.614 | 0.893 |
|  | rs2070970 | 59861983 | C/T | 0.98 | 0.560 | 0.561 | 0.550 | 0.564 |  | 1.00 ± 0.07 | 0.96 ± 0.09 | 1.01 ± 0.08 |  | 0.992 | 0.620 | 0.887 |
|  | rs2847668 | 59862261 | A/T | 0.98 | 0.560 | 0.562 | 0.551 | 0.564 |  | 1.00 ± 0.07 | 0.96 ± 0.09 | 1.01 ± 0.08 |  | 0.985 | 0.626 | 0.880 |
|  | rs569108 | 59863104 | A/G |  | 0.961 | 0.976 | 0.977 | 0.985 |  | 1.62 ± 0.21 | 1.76 ± 0.27 | 2.45 ± 0.29 |  | **0.012** ^d^ | **0.027** ^d^ | **2.7E-04** ^d^ |
|  | rs1290426 | 59864279 | C/G | 0.78 | 0.309 | 0.338 | 0.330 | 0.337 |  | 1.17 ± 0.09 | 1.13 ± 0.10 | 1.17 ± 0.10 |  | 0.058 | 0.235 | 0.107 |
|  | rs2847655 | 59865671 | T/C |  | 0.566 | 0.572 | 0.559 | 0.578 |  | 1.02 ± 0.07 | 0.97 ± 0.09 | 1.04 ± 0.08 |  | 0.818 | 0.685 | 0.619 |
|  | rs502419 | 59866175 | G/A | 0.99 | 0.520 | 0.536 | 0.529 | 0.547 |  | 1.07 ± 0.07 | 1.04 ± 0.09 | 1.12 ± 0.08 |  | 0.342 | 0.657 | 0.167 |
|  | rs2855017 | 59866309 | C/T | 0.99 | 0.559 | 0.560 | 0.551 | 0.564 |  | 1.00 ± 0.07 | 0.97 ± 0.09 | 1.02 ± 0.08 |  | 0.971 | 0.677 | 0.823 |
|  | rs17528859 | 59867379 | T/C | 0.98 | 0.559 | 0.560 | 0.551 | 0.564 |  | 1.00 ± 0.07 | 0.97 ± 0.09 | 1.02 ± 0.08 |  | 0.970 | 0.677 | 0.822 |
|  | rs574704 | 59867913 | A/G | 0.98 | 0.521 | 0.538 | 0.531 | 0.549 |  | 1.07 ± 0.07 | 1.04 ± 0.09 | 1.12 ± 0.08 |  | 0.330 | 0.654 | 0.157 |
| *IL4R* | rs2057768 | 27322095 | T/C |  | 0.306 | 0.309 | 0.313 | 0.311 |  | 1.01 ± 0.08 | 1.03 ± 0.09 | 1.03 ± 0.09 |  | 0.869 | 0.710 | 0.775 |
|  | rs2107356 | 27323404 | C/T | 0.90 | 0.601 | 0.603 | 0.593 | 0.588 |  | 1.01 ± 0.08 | 0.97 ± 0.09 | 0.95 ± 0.08 |  | 0.887 | 0.748 | 0.546 |
|  | rs8060938 | 27324881 | C/A | 0.93 | 0.556 | 0.562 | 0.551 | 0.550 |  | 1.03 ± 0.07 | 0.99 ± 0.09 | 0.99 ± 0.08 |  | 0.654 | 0.882 | 0.894 |
|  | rs12927172 | 27325021 | A/G | 0.93 | 0.399 | 0.408 | 0.409 | 0.407 |  | 1.04 ± 0.08 | 1.05 ± 0.09 | 1.04 ± 0.09 |  | 0.586 | 0.620 | 0.668 |
|  | rs12927543 | 27325023 | A/G | 0.90 | 0.897 | 0.895 | 0.901 | 0.897 |  | 0.97 ± 0.12 | 1.04 ± 0.15 | 0.99 ± 0.14 |  | 0.814 | 0.798 | 0.940 |
|  | rs34872711 | 27326206 | A/C | 0.89 | 0.849 | 0.850 | 0.862 | 0.860 |  | 1.00 ± 0.10 | 1.11 ± 0.13 | 1.09 ± 0.12 |  | 0.991 | 0.416 | 0.495 |
|  | rs7499292 | 27326268 | G/T | 0.93 | 0.399 | 0.408 | 0.409 | 0.407 |  | 1.04 ± 0.08 | 1.05 ± 0.09 | 1.04 ± 0.08 |  | 0.584 | 0.615 | 0.671 |
|  | rs8052962 | 27326842 | T/G | 0.93 | 0.399 | 0.408 | 0.409 | 0.407 |  | 1.04 ± 0.08 | 1.05 ± 0.09 | 1.04 ± 0.08 |  | 0.584 | 0.615 | 0.671 |
|  | rs11645013 | 27327116 | A/G | 0.95 | 0.554 | 0.564 | 0.554 | 0.551 |  | 1.05 ± 0.07 | 1.01 ± 0.09 | 1.00 ± 0.08 |  | 0.505 | 0.936 | 0.980 |
|  | rs8060025 | 27327214 | T/G | 0.93 | 0.399 | 0.408 | 0.409 | 0.407 |  | 1.04 ± 0.08 | 1.05 ± 0.09 | 1.04 ± 0.08 |  | 0.583 | 0.613 | 0.672 |
|  | rs8044444 | 27328543 | C/T | 0.88 | 0.845 | 0.845 | 0.856 | 0.858 |  | 0.99 ± 0.10 | 1.09 ± 0.13 | 1.09 ± 0.12 |  | 0.932 | 0.507 | 0.459 |
|  | rs7190969 | 27329118 | A/G | 0.90 | 0.897 | 0.895 | 0.900 | 0.897 |  | 0.97 ± 0.12 | 1.04 ± 0.15 | 0.99 ± 0.14 |  | 0.814 | 0.800 | 0.942 |
|  | rs1076238 | 27331030 | G/A | 0.91 | 0.608 | 0.610 | 0.600 | 0.594 |  | 1.01 ± 0.07 | 0.97 ± 0.09 | 0.94 ± 0.08 |  | 0.924 | 0.722 | 0.486 |
|  | rs6498011 | 27331894 | A/G | 0.95 | 0.400 | 0.411 | 0.411 | 0.409 |  | 1.05 ± 0.07 | 1.05 ± 0.09 | 1.04 ± 0.08 |  | 0.545 | 0.596 | 0.652 |
|  | rs6498012 | 27331974 | C/G | 0.95 | 0.400 | 0.411 | 0.411 | 0.409 |  | 1.05 ± 0.07 | 1.05 ± 0.09 | 1.04 ± 0.08 |  | 0.545 | 0.595 | 0.652 |
|  | rs7187471 | 27332334 | A/G | 0.95 | 0.400 | 0.411 | 0.411 | 0.409 |  | 1.05 ± 0.07 | 1.05 ± 0.09 | 1.04 ± 0.08 |  | 0.545 | 0.595 | 0.652 |
|  | rs34213105 | 27333381 | A/C | 0.92 | 0.851 | 0.854 | 0.865 | 0.864 |  | 1.01 ± 0.10 | 1.11 ± 0.13 | 1.09 ± 0.12 |  | 0.937 | 0.400 | 0.451 |
|  | rs35092916 | 27335002 | G/C | 0.92 | 0.851 | 0.854 | 0.865 | 0.864 |  | 1.01 ± 0.10 | 1.11 ± 0.13 | 1.09 ± 0.12 |  | 0.936 | 0.399 | 0.451 |
|  | rs1110470 | 27336427 | G/A |  | 0.547 | 0.558 | 0.549 | 0.542 |  | 1.04 ± 0.07 | 1.00 ± 0.09 | 1.00 ± 0.08 |  | 0.505 | 0.863 | 0.889 |
|  | rs78288118 | 27336437 | C/T | 0.97 | 0.552 | 0.561 | 0.551 | 0.549 |  | 1.04 ± 0.07 | 1.00 ± 0.09 | 1.00 ± 0.08 |  | 0.566 | 1.000 | 0.965 |
|  | rs71388093 | 27339024 | C/T | 0.94 | 0.307 | 0.323 | 0.332 | 0.322 |  | 1.08 ± 0.08 | 1.13 ± 0.10 | 1.07 ± 0.09 |  | 0.341 | 0.199 | 0.464 |
|  | rs35004258 | 27339027 | C/T | 0.94 | 0.307 | 0.323 | 0.332 | 0.322 |  | 1.08 ± 0.08 | 1.13 ± 0.10 | 1.07 ± 0.09 |  | 0.341 | 0.199 | 0.464 |
|  | rs9673499 | 27339145 | C/T | 0.94 | 0.307 | 0.323 | 0.332 | 0.322 |  | 1.08 ± 0.08 | 1.13 ± 0.10 | 1.07 ± 0.09 |  | 0.341 | 0.199 | 0.464 |
|  | rs58653621 | 27339762 | G/T | 0.94 | 0.465 | 0.479 | 0.475 | 0.465 |  | 1.06 ± 0.07 | 1.05 ± 0.09 | 1.00 ± 0.08 |  | 0.443 | 0.613 | 0.971 |
|  | rs6498013 | 27340735 | C/T | 0.93 | 0.309 | 0.322 | 0.331 | 0.321 |  | 1.07 ± 0.08 | 1.12 ± 0.10 | 1.06 ± 0.09 |  | 0.420 | 0.231 | 0.546 |
|  | rs4787948 | 27341059 | G/A | 0.96 | 0.301 | 0.315 | 0.325 | 0.314 |  | 1.07 ± 0.08 | 1.12 ± 0.10 | 1.06 ± 0.09 |  | 0.402 | 0.220 | 0.528 |
|  | rs7190472 | 27341553 | C/T | 0.97 | 0.305 | 0.320 | 0.329 | 0.318 |  | 1.08 ± 0.08 | 1.13 ± 0.09 | 1.06 ± 0.09 |  | 0.355 | 0.201 | 0.503 |
|  | rs34943813 | 27342379 | A/G | 0.93 | 0.852 | 0.856 | 0.866 | 0.864 |  | 1.02 ± 0.10 | 1.12 ± 0.13 | 1.10 ± 0.12 |  | 0.824 | 0.366 | 0.444 |
|  | rs78224329 | 27342381 | T/C | 0.92 | 0.854 | 0.858 | 0.868 | 0.867 |  | 1.03 ± 0.10 | 1.13 ± 0.13 | 1.10 ± 0.12 |  | 0.797 | 0.355 | 0.434 |
|  | rs12925861 | 27342596 | A/T | 0.97 | 0.305 | 0.320 | 0.330 | 0.318 |  | 1.08 ± 0.08 | 1.13 ± 0.09 | 1.06 ± 0.09 |  | 0.355 | 0.200 | 0.504 |
|  | rs2107355 | 27343479 | G/A | 0.90 | 0.314 | 0.331 | 0.343 | 0.328 |  | 1.09 ± 0.08 | 1.16 ± 0.10 | 1.07 ± 0.09 |  | 0.309 | 0.130 | 0.463 |
|  | rs2107354 | 27343515 | C/T | 0.97 | 0.305 | 0.320 | 0.330 | 0.318 |  | 1.08 ± 0.08 | 1.13 ± 0.09 | 1.06 ± 0.09 |  | 0.354 | 0.199 | 0.504 |
|  | rs4787951 | 27343963 | C/T | 0.96 | 0.301 | 0.317 | 0.328 | 0.314 |  | 1.08 ± 0.08 | 1.14 ± 0.09 | 1.06 ± 0.09 |  | 0.347 | 0.157 | 0.525 |
|  | rs35630199 | 27344199 | T/G | 0.96 | 0.843 | 0.846 | 0.860 | 0.855 |  | 1.01 ± 0.10 | 1.14 ± 0.12 | 1.08 ± 0.11 |  | 0.886 | 0.293 | 0.515 |
|  | rs3916997 | 27345114 | G/A | 0.98 | 0.464 | 0.477 | 0.473 | 0.463 |  | 1.06 ± 0.07 | 1.04 ± 0.09 | 1.00 ± 0.08 |  | 0.449 | 0.628 | 0.995 |
|  | rs1981551 | 27345352 | T/C | 0.96 | 0.301 | 0.317 | 0.328 | 0.314 |  | 1.08 ± 0.08 | 1.14 ± 0.09 | 1.06 ± 0.09 |  | 0.347 | 0.156 | 0.525 |
|  | rs2283563 | 27346354 | C/T |  | 0.672 | 0.703 | 0.708 | 0.700 |  | 1.16 ± 0.08 | 1.18 ± 0.09 | 1.14 ± 0.09 |  | **0.041** | 0.059 | 0.091 |
|  | rs12708699 | 27348165 | T/C | 0.97 | 0.846 | 0.849 | 0.861 | 0.859 |  | 1.01 ± 0.10 | 1.12 ± 0.12 | 1.09 ± 0.12 |  | 0.912 | 0.351 | 0.460 |
|  | rs12708700 | 27348322 | T/C | 0.97 | 0.846 | 0.849 | 0.861 | 0.859 |  | 1.01 ± 0.10 | 1.12 ± 0.12 | 1.09 ± 0.12 |  | 0.911 | 0.351 | 0.460 |
|  | rs12445247 | 27348630 | G/A | 0.95 | 0.557 | 0.567 | 0.560 | 0.553 |  | 1.05 ± 0.07 | 1.02 ± 0.09 | 0.99 ± 0.08 |  | 0.495 | 0.830 | 0.923 |
|  | rs3785356 | 27349168 | T/C | 0.96 | 0.301 | 0.316 | 0.328 | 0.313 |  | 1.08 ± 0.08 | 1.14 ± 0.09 | 1.06 ± 0.09 |  | 0.351 | 0.159 | 0.528 |
|  | rs9940480 | 27349438 | C/T | 0.97 | 0.457 | 0.469 | 0.466 | 0.457 |  | 1.05 ± 0.07 | 1.04 ± 0.09 | 1.01 ± 0.08 |  | 0.459 | 0.635 | 0.943 |
|  | rs9929928 | 27349493 | G/T | 0.98 | 0.847 | 0.851 | 0.862 | 0.860 |  | 1.02 ± 0.10 | 1.12 ± 0.12 | 1.09 ± 0.11 |  | 0.858 | 0.340 | 0.475 |
|  | rs2382720 | 27350012 | T/C | 0.98 | 0.463 | 0.474 | 0.471 | 0.462 |  | 1.05 ± 0.07 | 1.04 ± 0.09 | 1.00 ± 0.08 |  | 0.500 | 0.665 | 0.994 |
|  | rs3024530 | 27350687 | G/A | 0.95 | 0.471 | 0.478 | 0.476 | 0.467 |  | 1.03 ± 0.07 | 1.02 ± 0.09 | 0.99 ± 0.08 |  | 0.671 | 0.784 | 0.887 |
|  | rs2057767 | 27351338 | G/A | 0.95 | 0.471 | 0.477 | 0.476 | 0.467 |  | 1.03 ± 0.07 | 1.02 ± 0.09 | 0.99 ± 0.08 |  | 0.674 | 0.788 | 0.885 |
|  | rs3024536 | 27352713 | C/T | 0.99 | 0.848 | 0.852 | 0.864 | 0.860 |  | 1.02 ± 0.10 | 1.13 ± 0.12 | 1.09 ± 0.12 |  | 0.813 | 0.311 | 0.465 |
|  | rs3024537 | 27352819 | G/A |  | 0.847 | 0.852 | 0.863 | 0.860 |  | 1.02 ± 0.10 | 1.13 ± 0.12 | 1.09 ± 0.11 |  | 0.855 | 0.355 | 0.480 |
|  | rs3024543 | 27353230 | G/A | 0.99 | 0.848 | 0.853 | 0.864 | 0.861 |  | 1.03 ± 0.10 | 1.14 ± 0.12 | 1.09 ± 0.12 |  | 0.802 | 0.303 | 0.460 |
|  | rs3024544 | 27353357 | C/T | 0.99 | 0.848 | 0.853 | 0.864 | 0.861 |  | 1.03 ± 0.10 | 1.14 ± 0.12 | 1.09 ± 0.12 |  | 0.799 | 0.301 | 0.457 |
|  | rs3024546 | 27354067 | C/A | 0.99 | 0.847 | 0.853 | 0.864 | 0.861 |  | 1.03 ± 0.10 | 1.14 ± 0.12 | 1.09 ± 0.12 |  | 0.772 | 0.283 | 0.436 |
|  | rs3024547 | 27354361 | C/T | 0.99 | 0.847 | 0.853 | 0.864 | 0.861 |  | 1.03 ± 0.10 | 1.14 ± 0.12 | 1.09 ± 0.12 |  | 0.768 | 0.281 | 0.433 |
|  | rs3024548 | 27354531 | G/C | 0.98 | 0.462 | 0.471 | 0.468 | 0.459 |  | 1.04 ± 0.07 | 1.03 ± 0.09 | 0.99 ± 0.08 |  | 0.612 | 0.775 | 0.917 |
|  | rs3024550 | 27354947 | T/C | 0.99 | 0.847 | 0.853 | 0.865 | 0.861 |  | 1.03 ± 0.10 | 1.15 ± 0.12 | 1.10 ± 0.12 |  | 0.758 | 0.274 | 0.425 |
|  | rs3024552 | 27355004 | C/G | 0.98 | 0.458 | 0.466 | 0.463 | 0.456 |  | 1.04 ± 0.07 | 1.03 ± 0.09 | 1.00 ± 0.08 |  | 0.592 | 0.765 | 0.993 |
|  | rs3024554 | 27355285 | G/T | 0.98 | 0.847 | 0.853 | 0.865 | 0.861 |  | 1.03 ± 0.10 | 1.15 ± 0.12 | 1.10 ± 0.12 |  | 0.751 | 0.270 | 0.419 |
|  | rs3024555 | 27355349 | A/G | 0.98 | 0.847 | 0.853 | 0.865 | 0.861 |  | 1.03 ± 0.10 | 1.15 ± 0.12 | 1.10 ± 0.12 |  | 0.745 | 0.267 | 0.415 |
|  | rs3024556 | 27355362 | A/G | 0.97 | 0.304 | 0.318 | 0.327 | 0.316 |  | 1.07 ± 0.08 | 1.12 ± 0.09 | 1.05 ± 0.09 |  | 0.423 | 0.235 | 0.559 |
|  | rs1805010 | 27356203 | G/A |  | 0.458 | 0.447 | 0.429 | 0.433 |  | 1.03 ± 0.07 | 1.02 ± 0.09 | 0.99 ± 0.08 |  | 0.478 | 0.180 | 0.199 |
|  | rs2074572 | 27356359 | T/C | 0.96 | 0.364 | 0.387 | 0.395 | 0.386 |  | 1.10 ± 0.07 | 1.15 ± 0.09 | 1.10 ± 0.08 |  | 0.197 | 0.130 | 0.268 |
|  | rs2072130 | 27356398 | T/C | 0.97 | 0.368 | 0.391 | 0.396 | 0.391 |  | 1.10 ± 0.07 | 1.13 ± 0.09 | 1.10 ± 0.08 |  | 0.202 | 0.165 | 0.256 |
|  | rs3024560 | 27356667 | G/T | 0.96 | 0.367 | 0.393 | 0.399 | 0.393 |  | 1.12 ± 0.07 | 1.15 ± 0.09 | 1.12 ± 0.08 |  | 0.135 | 0.116 | 0.181 |
|  | rs2301807 | 27358098 | C/A |  | 0.962 | 0.974 | 0.971 | 0.976 |  | 1.41 ± 0.18 | 1.34 ± 0.22 | 1.50 ± 0.22 |  | 0.053 | 0.208 | **0.043** |
|  | rs3024575 | 27358132 | T/C | 0.96 | 0.381 | 0.415 | 0.420 | 0.417 |  | 1.15 ± 0.07 | 1.18 ± 0.09 | 1.16 ± 0.08 |  | 0.065 | 0.058 | 0.080 |
|  | rs3024577 | 27358203 | G/A | 0.98 | 0.475 | 0.500 | 0.505 | 0.501 |  | 1.11 ± 0.07 | 1.13 ± 0.09 | 1.11 ± 0.08 |  | 0.157 | 0.156 | 0.181 |
|  | rs2283562 | 27358763 | C/T | 0.96 | 0.472 | 0.491 | 0.490 | 0.483 |  | 1.08 ± 0.07 | 1.08 ± 0.09 | 1.05 ± 0.08 |  | 0.284 | 0.396 | 0.581 |
|  | rs2239348 | 27358948 | G/A | 0.98 | 0.566 | 0.577 | 0.574 | 0.568 |  | 1.05 ± 0.07 | 1.04 ± 0.09 | 1.02 ± 0.08 |  | 0.485 | 0.650 | 0.840 |
|  | rs2239347 | 27359021 | C/A | 0.96 | 0.467 | 0.485 | 0.485 | 0.479 |  | 1.08 ± 0.07 | 1.08 ± 0.09 | 1.05 ± 0.08 |  | 0.275 | 0.371 | 0.521 |
|  | rs2239346 | 27359061 | T/A | 0.96 | 0.467 | 0.485 | 0.485 | 0.479 |  | 1.08 ± 0.07 | 1.08 ± 0.09 | 1.05 ± 0.08 |  | 0.274 | 0.369 | 0.519 |
|  | rs3024585 | 27359844 | A/G |  | 0.473 | 0.496 | 0.499 | 0.504 |  | 1.10 ± 0.07 | 1.13 ± 0.09 | 1.11 ± 0.08 |  | 0.218 | 0.192 | 0.266 |
|  | rs3024586 | 27360041 | G/A |  | 0.956 | 0.963 | 0.951 | 0.966 |  | 1.16 ± 0.07 | 0.89 ± 0.09 | 1.28 ± 0.08 |  | 0.384 | 0.550 | 0.226 |
|  | rs3024589 | 27360627 | G/A | 0.88 | 0.499 | 0.514 | 0.519 | 0.511 |  | 1.07 ± 0.08 | 1.10 ± 0.09 | 1.06 ± 0.08 |  | 0.338 | 0.295 | 0.479 |
|  | rs3024590 | 27360628 | C/T | 0.88 | 0.493 | 0.509 | 0.515 | 0.508 |  | 1.08 ± 0.07 | 1.10 ± 0.09 | 1.07 ± 0.08 |  | 0.330 | 0.274 | 0.427 |
|  | rs2283561 | 27361167 | C/A |  | 0.424 | 0.455 | 0.462 | 0.458 |  | 1.11 ± 0.07 | 1.15 ± 0.09 | 1.11 ± 0.08 |  | 0.095 | 0.080 | 0.117 |
|  | rs3024607 | 27363611 | G/A |  | 0.916 | 0.929 | 0.932 | 0.939 |  | 1.16 ± 0.13 | 1.25 ± 0.17 | 1.34 ± 0.16 |  | 0.257 | 0.179 | **0.050** |
|  | rs3024610 | 27364158 | C/T | 0.99 | 0.497 | 0.514 | 0.514 | 0.511 |  | 1.06 ± 0.07 | 1.07 ± 0.09 | 1.05 ± 0.08 |  | 0.413 | 0.466 | 0.571 |
|  | rs3024611 | 27364200 | C/A | 0.97 | 0.844 | 0.864 | 0.877 | 0.878 |  | 1.18 ± 0.10 | 1.32 ± 0.13 | 1.32 ± 0.12 |  | 0.108 | **0.029** | **0.020** |
|  | rs3024613 | 27364253 | C/T |  | 0.500 | 0.482 | 0.486 | 0.514 |  | 1.06 ± 0.07 | 1.07 ± 0.09 | 1.05 ± 0.08 |  | 0.397 | 0.538 | 0.563 |
|  | rs3024619 | 27364806 | A/G | 0.98 | 0.342 | 0.379 | 0.391 | 0.389 |  | 1.16 ± 0.07 | 1.24 ± 0.09 | 1.21 ± 0.08 |  | **0.041** | **0.017** | **0.021** |
|  | rs3024622 | 27365453 | G/C | 0.98 | 0.342 | 0.379 | 0.391 | 0.389 |  | 1.16 ± 0.07 | 1.24 ± 0.09 | 1.21 ± 0.08 |  | **0.042** ^d^ | **0.017** ^d^ | **0.021** ^d^ |
|  | rs3024624 | 27365614 | A/G | 0.98 | 0.500 | 0.519 | 0.517 | 0.515 |  | 1.07 ± 0.07 | 1.07 ± 0.09 | 1.06 ± 0.08 |  | 0.321 | 0.429 | 0.501 |
|  | rs3024632 | 27366296 | T/C |  | 0.921 | 0.926 | 0.931 | 0.936 |  | 1.05 ± 0.13 | 1.17 ± 0.17 | 1.21 ± 0.16 |  | 0.820 | 0.430 | 0.237 |
|  | rs3024634 | 27366562 | A/G | 0.88 | 0.855 | 0.870 | 0.883 | 0.884 |  | 1.14 ± 0.11 | 1.31 ± 0.14 | 1.31 ± 0.13 |  | 0.227 | 0.053 | **0.035** |
|  | rs4787423 | 27367334 | T/C | 0.94 | 0.850 | 0.866 | 0.879 | 0.880 |  | 1.14 ± 0.11 | 1.30 ± 0.13 | 1.30 ± 0.12 |  | 0.208 | **0.046** | **0.036** |
|  | rs3024644 | 27367826 | G/C | 0.94 | 0.844 | 0.860 | 0.874 | 0.876 |  | 1.14 ± 0.10 | 1.30 ± 0.13 | 1.30 ± 0.12 |  | 0.202 | **0.043** | **0.030** |
|  | rs3024647 | 27367972 | A/G | 0.94 | 0.844 | 0.860 | 0.874 | 0.876 |  | 1.14 ± 0.10 | 1.30 ± 0.13 | 1.30 ± 0.12 |  | 0.203 | **0.044** | **0.029** |
|  | rs2891057 | 27368790 | T/A | 0.93 | 0.849 | 0.865 | 0.878 | 0.880 |  | 1.14 ± 0.11 | 1.30 ± 0.13 | 1.30 ± 0.12 |  | 0.217 | **0.050** | **0.036** |
|  | rs2891058 | 27368833 | A/G | 0.93 | 0.844 | 0.860 | 0.874 | 0.875 |  | 1.14 ± 0.10 | 1.30 ± 0.13 | 1.31 ± 0.12 |  | 0.210 | **0.046** | **0.029** |
|  | rs3024656 | 27369609 | G/A | 0.70 | 0.737 | 0.736 | 0.736 | 0.732 |  | 0.98 ± 0.10 | 0.99 ± 0.12 | 0.95 ± 0.11 |  | 0.801 | 0.911 | 0.642 |
|  | rs3024660 | 27371158 | T/C | 0.93 | 0.844 | 0.860 | 0.874 | 0.875 |  | 1.14 ± 0.10 | 1.30 ± 0.13 | 1.31 ± 0.12 |  | 0.211 | **0.047** | **0.029** |
|  | rs3024662 | 27371397 | G/A | 0.95 | 0.503 | 0.520 | 0.517 | 0.514 |  | 1.07 ± 0.07 | 1.06 ± 0.09 | 1.04 ± 0.08 |  | 0.385 | 0.508 | 0.610 |
|  | rs3024666 | 27371571 | A/G | 0.93 | 0.844 | 0.860 | 0.873 | 0.875 |  | 1.14 ± 0.10 | 1.29 ± 0.13 | 1.31 ± 0.12 |  | 0.216 | **0.049** | **0.029** |
|  | rs3024676 | 27373558 | C/A |  | 0.794 | 0.814 | 0.827 | 0.833 |  | 1.16 ± 0.09 | 1.25 ± 0.11 | 1.29 ± 0.10 |  | 0.121 | 0.054 | **0.013** |
|  | rs2234897 | 27373612 | C/T |  | 0.981 | 0.979 | 0.983 | 0.979 |  | 1.11 ± 0.07 | 0.91 ± 0.09 | 1.14 ± 0.10 |  | 0.667 | 0.767 | 0.653 |
|  | rs1805011 | 27373872 | A/C |  | 0.849 | 0.860 | 0.869 | 0.867 |  | 1.13 ± 0.10 | 1.20 ± 0.12 | 1.20 ± 0.12 |  | 0.306 | 0.195 | 0.172 |
|  | rs2234898 | 27373915 | G/T | 0.96 | 0.853 | 0.870 | 0.878 | 0.878 |  | 1.17 ± 0.10 | 1.24 ± 0.13 | 1.25 ± 0.12 |  | 0.124 | 0.088 | 0.061 |
|  | rs1805012 | 27373964 | T/C |  | 0.874 | 0.900 | 0.906 | 0.907 |  | 1.27 ± 0.11 | 1.33 ± 0.13 | 1.37 ± 0.13 |  | **0.018** ^d^ | **0.019** ^d^ | **0.008** ^d^ |
|  | rs2234900 | 27373972 | T/C | 0.97 | 0.848 | 0.878 | 0.882 | 0.888 |  | 1.31 ± 0.10 | 1.35 ± 0.13 | 1.43 ± 0.12 |  | **0.010** ^d^ | **0.020** ^d^ | **0.003** ^d^ |
|  | rs1805013 | 27373980 | C/T |  | 0.960 | 0.968 | 0.972 | 0.975 |  | 1.28 ± 0.07 | 1.43 ± 0.13 | 1.60± 0.13 |  | 0.188 | 0.131 | **0.040** ^d^ |
|  | rs1805015 | 27374180 | T/C |  | 0.809 | 0.846 | 0.859 | 0.864 |  | 1.28 ± 0.09 | 1.38 ± 0.12 | 1.45 ± 0.11 |  | **0.004** ^d^ | **0.002** ^d^ | **2.1E-04** ^d^ |
|  | rs1801275 | 27374400 | A/G |  | 0.765 | 0.793 | 0.803 | 0.807 |  | 1.20 ± 0.09 | 1.29 ± 0.11 | 1.33 ± 0.10 |  | **0.044** ^d^ | **0.034** ^d^ | **0.011** ^d^ |
|  | rs1805016 | 27374927 | T/G |  | 0.938 | 0.940 | 0.944 | 0.948 |  | 1.20 ± 0.16 | 1.35 ± 0.21 | 1.45 ± 0.20 |  | 0.822 | 0.582 | 0.321 |
|  | rs1049631 | 27375542 | G/A |  | 0.468 | 0.475 | 0.478 | 0.476 |  | 1.03 ± 0.07 | 1.05 ± 0.08 | 1.05 ± 0.08 |  | 0.771 | 0.663 | 0.778 |
|  | rs8832 | 27375787 | A/G | 0.96 | 0.462 | 0.471 | 0.474 | 0.473 |  | 1.04 ± 0.07 | 1.05 ± 0.09 | 1.05 ± 0.08 |  | 0.628 | 0.563 | 0.564 |
|  | rs1029489 | 27376217 | A/G | 0.97 | 0.390 | 0.408 | 0.415 | 0.413 |  | 1.08 ± 0.07 | 1.12 ± 0.09 | 1.11 ± 0.08 |  | 0.282 | 0.216 | 0.223 |
|  | rs2382721 | 27376637 | G/A | 0.93 | 0.346 | 0.368 | 0.382 | 0.380 |  | 1.11 ± 0.08 | 1.18 ± 0.09 | 1.17 ± 0.09 |  | 0.173 | 0.067 | 0.067 |
|  | rs3024685 | 27376910 | C/T | 0.94 | 0.392 | 0.412 | 0.420 | 0.417 |  | 1.09 ± 0.07 | 1.13 ± 0.09 | 1.12 ± 0.08 |  | 0.245 | 0.178 | 0.185 |
| *ADAM33* | rs512625 | 3648378 | G/A |  | 0.719 | 0.687 | 0.691 | 0.658 |  | 0.86 ± 0.08 | 0.88 ± 0.09 | 0.75 ± 0.13 |  | **0.047** | 0.147 | **0.031** |
|  | rs2787093 | 3648462 | T/C | 0.94 | 0.890 | 0.871 | 0.882 | 0.822 |  | 0.83 ± 0.11 | 0.92 ± 0.14 | 0.56 ± 0.17 |  | 0.080 | 0.537 | **4.9E-04** |
|  | rs614971 | 3648850 | G/C | 0.92 | 0.692 | 0.677 | 0.679 | 0.677 |  | 0.94 ± 0.08 | 0.94 ± 0.10 | 0.94 ± 0.14 |  | 0.436 | 0.538 | 0.670 |
|  | rs517155 | 3648877 | G/A | 0.75 | 0.899 | 0.904 | 0.906 | 0.894 |  | 1.09 ± 0.14 | 1.12 ± 0.17 | 0.92 ± 0.23 |  | 0.547 | 0.521 | 0.714 |
|  | rs2787094 | 3649161 | G/C | 0.86 | 0.808 | 0.784 | 0.792 | 0.762 |  | 0.85 ± 0.09 | 0.89 ± 0.11 | 0.73 ± 0.15 |  | 0.070 | 0.302 | **0.039** ^d^ |
|  | rs677044 | 3649431 | A/G | 0.93 | 0.754 | 0.746 | 0.738 | 0.744 |  | 0.95 ± 0.08 | 0.91 ± 0.10 | 0.94 ± 0.14 |  | 0.556 | 0.358 | 0.677 |
|  | rs543749 | 3649679 | G/T | 0.96 | 0.892 | 0.879 | 0.873 | 0.912 |  | 0.88 ± 0.11 | 0.82 ± 0.13 | 1.27 ± 0.22 |  | 0.248 | 0.145 | 0.272 |
|  | rs628965 | 3649713 | G/A | 0.99 | 0.619 | 0.604 | 0.606 | 0.523 |  | 0.93 ± 0.07 | 0.94 ± 0.09 | 0.65 ± 0.13 |  | 0.299 | 0.485 | **0.001** ^d^ |
|  | rs628977 | 3649721 | C/T |  | 0.617 | 0.600 | 0.603 | 0.516 |  | 0.93 ± 0.07 | 0.94 ± 0.09 | 0.66 ± 0.13 |  | 0.231 | 0.455 | **3.7E-04** ^d^ |
|  | rs678881 | 3649803 | G/C |  | 0.729 | 0.730 | 0.721 | 0.700 |  | 1.00 ± 0.08 | 0.96 ± 0.10 | 0.85 ± 0.13 |  | 0.966 | 0.664 | 0.249 |
|  | rs630712 | 3650066 | A/C | 0.97 | 0.892 | 0.874 | 0.886 | 0.826 |  | 0.84 ± 0.11 | 0.94 ± 0.14 | 0.57 ± 0.17 |  | 0.102 | 0.620 | **0.001** |
|  | rs2280089 | 3650127 | G/A | 0.99 | 0.873 | 0.869 | 0.877 | 0.847 |  | 0.99 ± 0.10 | 1.05 ± 0.13 | 0.83 ± 0.17 |  | 0.884 | 0.692 | 0.277 |
|  | rs2280090 | 3650205 | G/A |  | 0.876 | 0.876 | 0.887 | 0.857 |  | 0.99 ± 0.10 | 1.05 ± 0.13 | 0.83 ± 0.17 |  | 0.816 | 0.390 | 0.440 |
|  | rs2280091 | 3650234 | A/G |  | 0.868 | 0.858 | 0.859 | 0.841 |  | 0.99 ± 0.10 | 1.05 ± 0.13 | 0.83 ± 0.17 |  | 0.547 | 0.616 | 0.258 |
|  | rs574174 | 3650694 | C/T |  | 0.818 | 0.807 | 0.797 | 0.829 |  | 0.94 ± 0.09 | 0.88 ± 0.11 | 1.08 ± 0.16 |  | 0.443 | 0.203 | 0.585 |
|  | rs597980 | 3651165 | A/G | 0.96 | 0.440 | 0.415 | 0.422 | 0.326 |  | 0.90 ± 0.07 | 0.93 ± 0.09 | 0.60 ± 0.14 |  | 0.143 | 0.399 | **1.2E-04** ^d^ |
|  | rs44707 | 3651226 | T/G | 0.95 | 0.573 | 0.550 | 0.549 | 0.483 |  | 0.90 ± 0.07 | 0.90 ± 0.09 | 0.68 ± 0.13 |  | 0.146 | 0.231 | **0.002** ^d^ |
|  | rs598418 | 3651269 | A/G | 0.98 | 0.618 | 0.603 | 0.605 | 0.523 |  | 0.93 ± 0.07 | 0.94 ± 0.09 | 0.65 ± 0.13 |  | 0.305 | 0.478 | **0.001** ^d^ |
|  | rs2853209 | 3651472 | A/T | 0.93 | 0.482 | 0.465 | 0.474 | 0.362 |  | 0.93 ± 0.07 | 0.97 ± 0.09 | 0.58 ± 0.13 |  | 0.321 | 0.719 | **3.8E-05** ^d^ |
|  | rs528557 | 3651742 | C/G |  | 0.737 | 0.721 | 0.722 | 0.728 |  | 0.93 ± 0.08 | 0.93 ± 0.10 | 0.99 ± 0.14 |  | 0.411 | 0.476 | 0.926 |
|  | rs612709 | 3652207 | G/A | 0.96 | 0.868 | 0.850 | 0.844 | 0.878 |  | 0.87 ± 0.10 | 0.82 ± 0.12 | 1.12 ± 0.19 |  | 0.165 | 0.106 | 0.555 |
|  | rs3918395 | 3653149 | C/A | 0.97 | 0.869 | 0.868 | 0.877 | 0.850 |  | 1.02 ± 0.10 | 1.09 ± 0.13 | 0.89 ± 0.17 |  | 0.842 | 0.504 | 0.499 |
|  | rs2280092 | 3653587 | C/T | 0.97 | 0.869 | 0.868 | 0.877 | 0.850 |  | 1.02 ± 0.10 | 1.09 ± 0.13 | 0.89 ± 0.17 |  | 0.843 | 0.504 | 0.499 |
|  | rs2280094 | 3653709 | C/A | 0.92 | 0.768 | 0.770 | 0.753 | 0.772 |  | 1.00 ± 0.09 | 0.91 ± 0.10 | 0.99 ± 0.15 |  | 0.956 | 0.354 | 0.966 |
|  | rs2271511 | 3654433 | C/T | 0.93 | 0.802 | 0.788 | 0.798 | 0.780 |  | 0.94 ± 0.09 | 0.99 ± 0.11 | 0.92 ± 0.15 |  | 0.489 | 0.908 | 0.570 |
|  | rs2485700 | 3654993 | T/C | 0.87 | 0.838 | 0.826 | 0.829 | 0.831 |  | 0.92 ± 0.10 | 0.94 ± 0.12 | 0.97 ± 0.17 |  | 0.410 | 0.586 | 0.873 |
|  | rs511898 | 3655085 | T/C |  | 0.641 | 0.625 | 0.632 | 0.373 |  | 1.06 ± 0.07 | 1.04 ± 0.09 | 1.05 ± 0.13 |  | 0.480 | 0.730 | 0.806 |
|  | rs2787095 | 3655943 | C/G |  | 0.584 | 0.542 | 0.568 | 0.530 |  | 1.16 ± 0.07 | 1.06 ± 0.09 | 1.51 ± 0.12 |  | **0.027** ^d^ | 0.485 | **3.8E-04** ^d^ |
|  | rs17548927 | 3657387 | G/A | 0.69 | 0.858 | 0.856 | 0.849 | 0.863 |  | 0.97 ± 0.12 | 0.90 ± 0.14 | 1.06 ± 0.21 |  | 0.814 | 0.461 | 0.799 |
|  | rs568251 | 3657803 | G/A | 0.95 | 0.867 | 0.868 | 0.858 | 0.859 |  | 1.02 ± 0.11 | 0.93 ± 0.13 | 0.95 ± 0.18 |  | 0.858 | 0.538 | 0.777 |
|  | rs597165 | 3657804 | A/T |  | 0.864 | 0.864 | 0.855 | 0.854 |  | 1.02 ± 0.11 | 0.93 ± 0.12 | 0.95 ± 0.18 |  | 0.956 | 0.551 | 0.738 |
|  | rs2853210 | 3658211 | C/T |  | 0.760 | 0.763 | 0.772 | 0.709 |  | 1.02 ± 0.09 | 1.07 ± 0.11 | 0.76 ± 0.15 |  | 0.720 | 0.494 | 0.092 |
|  | rs487377 | 3658931 | T/C | 0.70 | 0.190 | 0.209 | 0.206 | 0.249 |  | 1.19 ± 0.11 | 1.16 ± 0.13 | 1.66 ± 0.18 |  | 0.109 | 0.258 | **0.004** |
|  | rs570269^e^ | 3659647 | G/C |  | 0.824 | 0.809 | 0.818 | 0.717 |  | 0.85 ± 0.12 | 0.93 ± 0.14 | 0.57 ± 0.19 |  | 0.086 | 0.287 | **0.005** |
|  | rs603112 | 3660789 | C/T | 0.67 | 0.853 | 0.843 | 0.847 | 0.804 |  | 0.87 ± 0.12 | 0.93 ± 0.15 | 0.59 ± 0.19 |  | 0.255 | 0.595 | **0.005** |
|  | rs2853211 | 3660947 | C/G | 0.54 | 0.279 | 0.279 | 0.277 | 0.294 |  | 0.99 ± 0.11 | 0.98 ± 0.13 | 1.13 ± 0.18 |  | 0.954 | 0.896 | 0.488 |
|  | rs2595584 | 3661029 | C/G | 0.55 | 0.409 | 0.426 | 0.423 | 0.459 |  | 1.15 ± 0.10 | 1.12 ± 0.12 | 1.48 ± 0.16 |  | 0.136 | 0.327 | **0.017** |
|  | rs2853212 | 3661550 | A/G | 0.70 | 0.535 | 0.546 | 0.540 | 0.562 |  | 1.09 ± 0.09 | 1.04 ± 0.10 | 1.20 ± 0.15 |  | 0.333 | 0.687 | 0.212 |
|  | rs17548962 | 3661803 | C/T |  | 0.967 | 0.960 | 0.962 | 0.951 |  | 0.82 ± 0.19 | 0.85 ± 0.23 | 0.67 ± 0.30 |  | 0.289 | 0.534 | 0.205 |
|  | rs2853213 | 3661840 | C/G |  | 0.655 | 0.667 | 0.664 | 0.658 |  | 1.08 ± 0.08 | 1.05 ± 0.09 | 1.06 ± 0.13 |  | 0.319 | 0.580 | 0.779 |
|  | rs554743 | 3662142 | T/C |  | 0.733 | 0.724 | 0.724 | 0.701 |  | 0.95 ± 0.08 | 0.95 ± 0.10 | 0.86 ± 0.13 |  | 0.442 | 0.566 | 0.215 |
|  | rs553863 | 3662235 | A/C | 0.81 | 0.606 | 0.618 | 0.618 | 0.630 |  | 1.08 ± 0.08 | 1.07 ± 0.10 | 1.16 ± 0.14 |  | 0.327 | 0.484 | 0.293 |
|  | rs2853214 | 3662272 | C/T | 0.79 | 0.613 | 0.626 | 0.626 | 0.639 |  | 1.10 ± 0.08 | 1.09 ± 0.10 | 1.19 ± 0.14 |  | 0.274 | 0.399 | 0.238 |
|  | rs8124875 | 3663553 | T/G | 0.67 | 0.352 | 0.354 | 0.354 | 0.343 |  | 1.02 ± 0.09 | 1.01 ± 0.11 | 0.95 ± 0.15 |  | 0.846 | 0.908 | 0.746 |
| tSNPs are underlined. Nominal significant associations in bold. NA: not available because the gene where it resides did not show any SNP at nominal significance or was not followed in this analysis.  ^a^According to NCBI build 36.3; ^b^Squared correlation between true genotypes and estimated allelic dosage of imputed SNPs. Note that the measure is not reported for tSNPs; ^c^Computed for allele 1; ^d^SNPs associated in previous studies; ^e^No distinction was made between C and the new T allele for this analysis. | | | | | | | | | | | | | | | | |
