# Supplementary material for: Assessing the Validity of Asthma Associations for Eight Candidate Genes and Age at Diagnosis Effects
Source: PLoS One. 2013 Sep 9;8(9):e73157. doi: 10.1371/journal.pone.0073157 (PMC3767824; doi:10.1371/journal.pone.0073157)
Supplement: Table S4 — Functional annotation of the 10 associated SNPs. (DOC) [file pone.0073157.s004.doc]

| **Table S4.** Functional annotation of the 10 associated SNPs | | | | | |
| --- | --- | --- | --- | --- | --- |
| Gene | rs# | dbSNP annotation | Proteins bound | Promoter histone marks | DNAse positive |
| *MS4A2* | rs569108 | Missense | USF1 | - | - |
| *IL4R* | rs1805015 | Missense | - | - | - |
| *ADAM33* | rs2787093 | 3' UTR | - | - | - |
|  | rs628965 | Intronic | - | - | - |
|  | rs628977 | Intronic | - | - | - |
|  | rs630712 | Intronic | - | - | - |
|  | rs597980 | Intronic | CTCF | - | Fibrobl |
|  | rs598418 | Intronic | CTCF,RAD21 | HepG2 | 28 cell types^a^ |
|  | rs2853209 | Intronic | CTCF | HepG2 | Caco-2 |
|  | rs2787095 | Intronic | ZNF263 | - | - |
| ^a^H1-hESC, HSMM, HSMMtube, HUVEC, LNCaP, MCF-7, 8988T, Fibrobl, GM12891, Gliobla, Ishikawa, Melano, Myometr, Osteobl, PanIsletD, Stellate, Urothelia, AG09309, BE2_C, H7-hESC, HCT-116, HFF, HFF-Myc, HMF, NHDF-Ad, NHDF-neo, NT2-D1, WI-38. | | | | | |
