# Supplementary material for: Assessing the Validity of Asthma Associations for Eight Candidate Genes and Age at Diagnosis Effects
Source: PLoS One. 2013 Sep 9;8(9):e73157. doi: 10.1371/journal.pone.0073157 (PMC3767824; doi:10.1371/journal.pone.0073157)
Supplement: Table S5 — In silico replication of the associated SNPs contained in the GABRIEL study. (DOC) [file pone.0073157.s005.doc]

| **Table S4.** *In silico* replication of the 11 associated SNPs contained in the GABRIEL study (10,365 cases and 16,110 controls). | | | | | |
| --- | --- | --- | --- | --- | --- |
| Gene | SNP | position | Reference allele | OR (95% CI) | *p*-value |
| *LTA-TNF* | rs2009658 | 31646223 | C | 1.00 (0.94-1.06) | 0.863 |
|  | rs2844482 | 31647746 | C | 0.99 (0.94-1.05) | 0.754 |
|  | rs2229094 | 31648535 | C | 1.03 (0.99-1.08) | 0.170 |
| *IL4R* | rs2283563 | 27253855 | C | 0.96 (0.92-1.00) | 0.064 |
|  | rs3024619 | 27272307 | A | 0.97 (0.93-1.01) | 0.107 |
|  | rs1805012 | 27281465 | C | 1.12 (1.05-1.19) | 5.73E-04 |
|  | rs1805015 | 27281681 | C | 1.04 (0.98-1.09) | 0.181 |
|  | rs1801275 | 27281901 | A | 0.97 (0.93-1.02) | 0.244 |
|  | rs3024660 | 27278659 | C | 1.02 (0.97-1.08) | 0.402 |
| *ADAM33* | rs512625 | 3596378 | A | 0.97 (0.93-1.01) | 0.105 |
|  | rs487377 | 3606931 | C | 1.03 (0.98-1.08) | 0.240 |
|  | | | | | |
