# Supplementary material for: Assessing the Validity of Asthma Associations for Eight Candidate Genes and Age at Diagnosis Effects
Source: PLoS One. 2013 Sep 9;8(9):e73157. doi: 10.1371/journal.pone.0073157 (PMC3767824; doi:10.1371/journal.pone.0073157)
Supplement: Table S6 — Sample sizes and statistical power for each analysis performed in a subset of cases. (DOC) [file pone.0073157.s006.doc]

| **Table S5.** Sample sizes and statistical power for each analysis performed in a subset of cases. | | | |
| --- | --- | --- | --- |
| Subset of analysis | Number of cases | Statistical power MAF=0.20^a^ | Statistical power MAF=0.45^b^ |
| Asthma | 606 | 48.4% | 69.6% |
| Atopic | 356 | 29.3% | 44.1% |
| Asthma with age-at-diagnosis ≤ 14 | 155 | 10.8% | 14.6% |
| Asthma with age-at-diagnosis ≤ 26 | 291 | 23.4% | 35.0% |
| Asthma with age-at-diagnosis ≤ 39 | 427 | 35.4% | 52.6% |
| ^a^Assessed to detect a minimum risk of 1.45 and a two-sided *p*=0.0012 significance level for a minor allele frequency of 20%^a^ and a minor allele frequency of 45%^b^. | | | |
