# Supplementary material for: Assessing the Validity of Asthma Associations for Eight Candidate Genes and Age at Diagnosis Effects
Source: PLoS One. 2013 Sep 9;8(9):e73157. doi: 10.1371/journal.pone.0073157 (PMC3767824; doi:10.1371/journal.pone.0073157)
Supplement: Table S7 — Coverage of candidate genes on commercial arrays used in asthma GWAS in samples of European ancestry. (DOC) [file pone.0073157.s007.doc]

| **Table S6.** Coverage of candidate genes on commercial arrays used in asthma GWAS in samples of European ancestry.^a^ | | | | | | | | | | |
| --- | --- | --- | --- | --- | --- | --- | --- | --- | --- | --- |
|  |  | HapMap CEU | | | |  | 1KGP CEU | | | |
| Gene | SNP Array | SNPs MAF>5% | SNPs in array | # tagged SNPs^b^ | Coverage (%) |  | SNPs MAF>5% | SNPs in array | # tagged SNPs^b^ | Coverage (%) |
| *IL13-IL4* | Affymetrix 500K | 34 | 11 | 30 | 88 |  | 47 | 7 | 36 | 77 |
|  | Affymetrix Genome-Wide Human SNP Array 6.0 | 34 | 11 | 30 | 88 |  | 47 | 10 | 37 | 79 |
|  | Illumina HumanHap 300^c^ | 34 | 5 | 14 | 41 |  | 47 | 4 | 17 | 36 |
|  | Illumina HumanHap 550^d^ | 34 | 7 | 24 | 71 |  | 47 | 7 | 32 | 68 |
|  | Illumina HumanHap 650 | 34 | 12 | 28 | 82 |  | 47 | 9 | 32 | 68 |
|  | Illumina HumanHap I1M-Duo | 34 | 33 | 34 | 100 |  | 47 | 40 | 40 | 85 |
| *CD14* | Affymetrix 500K | 6 | 2 | 5 | 83 |  | 15 | 2 | 13 | 87 |
|  | Affymetrix Genome-Wide Human SNP Array 6.0 | 6 | 3 | 5 | 83 |  | 15 | 3 | 13 | 87 |
|  | Illumina HumanHap 300^c^ | 6 | 0 | 0 | 0 |  | 15 | 0 | 0 | 0 |
|  | Illumina HumanHap 550^d^/650 | 6 | 1 | 3 | 50 |  | 15 | 1 | 4 | 27 |
|  | Illumina HumanHap I1M-Duo | 6 | 2 | 3 | 50 |  | 15 | 2 | 4 | 27 |
| *ADRB2* | Affymetrix 500K, Affymetrix Genome-Wide Human SNP Array 6.0 | 10 | 2 | 7 | 70 |  | 27 | 18 | 17 | 63 |
|  | Illumina HumanHap 300^c^ | 10 | 3 | 7 | 70 |  | 27 | 3 | 4 | 15 |
|  | Illumina HumanHap 550^d^ | 10 | 4 | 7 | 70 |  | 27 | 5 | 21 | 78 |
|  | Illumina HumanHap 650 | 10 | 5 | 9 | 90 |  | 27 | 7 | 23 | 85 |
|  | Illumina HumanHap I1M-Duo | 10 | 10 | 10 | 100 |  | 27 | 18 | 25 | 93 |
| *LTA-TNF* | Affymetrix 500K, Affymetrix Genome-Wide Human SNP Array 6.0 | 13 | 1 | 1 | 8 |  | 25 | 1 | 1 | 4 |
|  | Illumina HumanHap 300^c^/550^d^/650 | 13 | 5 | 8 | 62 |  | 25 | 5 | 14 | 56 |
|  | Illumina HumanHap I1M-Duo | 13 | 13 | 13 | 100 |  | 25 | 20 | 20 | 80 |
| *MS4A2* | Affymetrix 500K | 21 | 2 | 20 | 95 |  | 34 | 2 | 24 | 71 |
|  | Affymetrix Genome-Wide Human SNP Array 6.0 | 21 | 3 | 20 | 95 |  | 34 | 3 | 24 | 71 |
|  | Illumina HumanHap 300^c^ | 21 | 0 | 0 | 0 |  | 34 | 0 | 0 | 0 |
|  | Illumina HumanHap 550^d^/650 | 21 | 3 | 20 | 95 |  | 34 | 3 | 24 | 71 |
|  | Illumina HumanHap I1M | 21 | 9 | 21 | 100 |  | 34 | 8 | 31 | 91 |
| *IL4R* | Affymetrix 500K | 55 | 11 | 19 | 35 |  | 143 | 10 | 56 | 39 |
|  | Affymetrix Genome-Wide Human SNP Array 6.0 | 55 | 20 | 39 | 71 |  | 143 | 24 | 110 | 77 |
|  | Illumina HumanHap 300^c^ | 55 | 12 | 35 | 64 |  | 143 | 12 | 86 | 60 |
|  | Illumina HumanHap 550^d^ | 55 | 20 | 51 | 93 |  | 143 | 18 | 115 | 80 |
|  | Illumina HumanHap 650 | 55 | 26 | 53 | 96 |  | 143 | 22 | 128 | 90 |
|  | Illumina HumanHap I1M-Duo | 55 | 47 | 54 | 98 |  | 143 | 81 | 138 | 97 |
| *ADAM33* | Affymetrix 500K Affymetrix Genome-Wide Human SNP Array 6.0 | 17 | 2 | 4 | 24 |  | 50 | 2 | 4 | 8 |
|  | Illumina HumanHap 300^c^ | 17 | 3 | 3 | 18 |  | 50 | 3 | 3 | 6 |
|  | Illumina HumanHap 550^d^/650 | 17 | 5 | 5 | 29 |  | 50 | 5 | 9 | 18 |
|  | Illumina HumanHap I1M-Duo | 17 | 11 | 13 | 76 |  | 50 | 11 | 14 | 28 |
| ^a^Includes studies described in Moffatt et al. 2007, Himes et al. 2009, Sleiman et al. 2009, Li et al. 2010, Moffatt et al. 2010, Ferreira et al. 2011, Torgerson et al. 2011.  ^b^SNPs captured by the array both directly and indirectly (with a multi-marker *r*^2^≥0.8)  ^c^Including the Illumina HumanCNV370-Duo as it was built upon the Illumina HumanHap300 BeadChip (317K SNPs) with additional 52K markers aimed at detecting CNVs.  ^d^The Illumina Human610 SNP content is similar to the Illumina HumanHap550 with the addition of 60K markers to target regions with CNVs. | | | | | | | | | | |
