# Supplementary material for: Assessing the Validity of Asthma Associations for Eight Candidate Genes and Age at Diagnosis Effects
Source: PLoS One. 2013 Sep 9;8(9):e73157. doi: 10.1371/journal.pone.0073157 (PMC3767824; doi:10.1371/journal.pone.0073157)
Supplement: Text S1 — Supplementary methods. (DOC) [file pone.0073157.s008.doc]

**Text S1. Supplementary methods**

**Study subjects**

This study was conducted using a case-control design of 1,878 DNA samples from unrelated individuals, all reporting at least two generations of Spanish descent. Sample details have been described elsewhere .

Cases included 607 asthmatic patients aged >5 years and diagnosed by physicians following the Global Initiative for Asthma (GINA) guidelines for diagnosis and classification of asthma severity (http://www.ginasthma.com). These samples were collected and characterized for allergic and asthmatic symptoms in Respiratory Medicine and Allergy Departments, as part of the Genetics of Asthma study (GOA) in the Spanish population. Among cases, atopy was defined by the evidence of allergic sensitization to known allergens, reflected by either a positive skin prick test [SPT] or the specific IgE to one or more known allergens in the serum. These tests evaluated seven common allergen collections: dust mites (*Dermatophagoides pteronyssinus, Dermatophagoides farinae, Glycyphagus spp, Blomia tropicalis, Acarus siro, Tyrophagus putrescentiae, Lepidoglyphus destructor, Euroglyphus maynei*), epithelia (*Felis domesticus, Canis familiaris, Equus caballus, Oryctolagus cuniculus*), pollen (*Olea europaea, Salsola spp, Lolium perenne, Artemisia vulgaris*, *Parietaria* spp, *Platanus* spp, *Chenopodium* spp, *Plantago* spp, *Rumex* spp, *Cupressus* spp), fungi (*Penicillium notatum, Alternaria alternate, Aspergillus fumigatus, Cladosporium herbarum*), food (Cow's milk, hen's egg, peanut, soybean, wheat, fish, crustaceans [shrimp, crabs, and lobster], mollusks [clams, oysters, and mussels], banana, chestnut, kiwi), latex, and others (*Blattella germanica*). The SPT was considered positive when the individual had a wheal with a diameter 3 mm greater than the saline control. In addition, specific IgE levels were measured in asthmatic individuals, allowing to classify them in those with a positive result to, at least, one of seven common allergens (specific IgE levels ≥ 0.35 UI mL-1) and those with a negative test (<0.35 UI mL-1). For simplicity, those cases that had asthma and also atopy will be referred as atopic asthmatics, although we ignored whether or not allergen exposures lead to the asthma symptoms of these patients. Further sample details can be found in Table S1.

Control group consisted of 1,271 DNA samples from adults self-reporting no personal or familiar medical history of allergic or pulmonary diseases from the Spanish National DNA Biobank. These were collected from branches of the National Blood Service from unrelated individuals residing in Spain. After signed informed consent, by means of personal interviews, each donor was asked to declare general health status, physic activity, commonly used transportation, nutrition habits, type of work and qualification, demographics, tobacco smoke, alcohol consumption, genealogical information, residence and mother tongue, and personal and familial history of diseases including infectious, cancerous, blood and circulatory, endocrine, mental or behavioral, nervous, vision, hearing, respiratory (including asthma symptoms), immunological (including allergies), bone, congenital, skin and digestive. No immediate family (parents, brothers or sisters, children) that has donated samples to the Spanish National DNA Biobank could be a DNA sample donor. The Spanish National DNA Biobank criteria for sample classification as healthy was based on self-reported absence of cardiovascular, renal, pulmonary, hepatic, hematological illnesses or any other chronic conditions which require continuous treatment, hepatitis B, C infections or acquired immunodeficiency syndrome (AIDS), However, no clinical characterization was performed on any subject, no information from medical record was incorporated, and no medical testing was performed on these individuals. For further information visit http://www.bancoadn.org. In addition to the criteria of the Spanish National DNA Biobank to define healthy, we added three more criteria to select the controls for this study: 1) Self-reported Spanish ancestry based on having at least two generations of ancestors born in Spain; 2) Complete data on personal and familiar history of disease recorded in the questionnaire, smoking status, place of origin, and area of residence; 3) Absence of self-reported personal or familiar history of pulmonary or allergic disease.

**Selection of tagging SNPs**

Tagging SNPs (tSNPs) were selected by means of TagIT 3.03 , using available re-sequencing data from European samples from different sources (Table 1 and Table S2). It is worth noting that the re-sequencing data for the *MS4A2* gene was unavailable from any public database when the study started. Thus, for this gene, tSNP selection was done using data from 60 unrelated samples from Utah residents with ancestry from northern and western Europe (CEU) from HapMap and tagging performance was verified against re-sequencing data from 96 type 1 diabetes individuals of white European ancestry (v0.1, accessed on 19^th^ March 2008) . The *IL13* and *IL4* genes, which lie in close proximity, were considered as a single region. Similarly, given the strong linkage disequilibrium (LD) between *LTA* and *TNF* genes , common variants of the *LTA* gene were also tagged and jointly analyzed with *TNF*. In all cases, CEU genotypes were also used to complement the set of tSNPs selected from all genes to ensure adequate gene coverage. In addition, previously associated SNPs and all potential functional variants as predicted by PupaSuite were forced to be included as tSNPs. The final tSNPs set was further tested for tagging performance using a SNP-dropping-with-resampling method , satisfying a haplotype *r^2^*≥0.85 in the reference genotype data. See Table S2 for further details.

**Genotyping**

DNA was extracted from blood or saliva and used for whole genome amplification with Illustra *GenomiPhi* V2 DNA Amplification Kit (GE Healthcare, Little Chalfont, UK) as described elsewhere . Genotyping was conducted using the iPLEX® Gold assay on MassARRAY® system (Sequenom Inc., San Diego, CA) by the Spanish National Genotyping Center, Santiago de Compostela Node (CeGen, http://www.cegen.org). Briefly, iPLEX® assays were scanned by MALDI-TOF mass spectrometry and individual SNP genotype calls were automatically generated using Sequenom TYPER 3.4® software (Sequenom Inc.). Samples from the Coriell Institute for Medical Research (http://www.coriell.org) were included on each SpectroCHIP® (Sequenom Inc.) to test allele calling reliability samples of this platform.

The SNPs that gave poor quality data on iPLEX® Gold assay on MassARRAY® system were finally determined at the Hospital Universitario N. S. de Candelaria using either SNaPshot® Multiplex kit reactions (Applied Biosystems, Foster City, CA) or KASPar SNP Genotyping System assays (KBiosciences, Hertfordshire, UK). For that, we alternatively utilized an ABI 3500 Genetic Analyzer (Applied Biosystems), automatically assigning genotypes using GeneMapper v3.2 (Applied Biosystems), or a 7500 Fast Real-Time PCR System (Applied Biosystems), with automated calls generated using the 7500 software 2.0.1 based on discriminating plots with 95% confidence, respectively. Genotyping was blind to the disease status and ≈6% of the samples were genotyped in duplicate to monitor genotyping quality.

Contrary to the dbSNPs database, that defined the SNP rs570269 in *ADAM33* gene as a C/G change, this SNP was detected as a triallelic *locus* during genotyping with iPLEX® Gold assays. This information has been submitted to NCBI (pending on the submission number) as it was confirmed by sequencing of individuals showing the new T allele (0.4% of controls) by both strands with BigDye® v3.1 Terminator Cycle Sequencing kit (Applied Biosystems) under the manufacturer’s recommendations and using the amplification primers. Sequencing products were ethanol precipitated and run on an ABI 3500 Genetic Analyzer. However, no distinction was made between T and C alleles during the statistical analysis of association.

**Supplementary references**

1. Pino-Yanes M, Sanchez-Machin I, Cumplido J, Figueroa J, Torres-Galvan MJ, et al. (2012) IL-1 receptor-associated kinase 3 gene (*IRAK3*) variants associate with asthma in a replication study in the Spanish population. J Allergy Clin Immunol 129: 573-575 e510.

2. Ahmadi KR, Weale ME, Xue ZY, Soranzo N, Yarnall DP, et al. (2005) A single-nucleotide polymorphism tagging set for human drug metabolism and transport. Nat Genet 37: 84-89.

3. Frazer KA, Ballinger DG, Cox DR, Hinds DA, Stuve LL, et al. (2007) A second generation human haplotype map of over 3.1 million SNPs. Nature 449: 851-861.

4. Smyth DJ, Howson JM, Payne F, Maier LM, Bailey R, et al. (2006) Analysis of polymorphisms in 16 genes in type 1 diabetes that have been associated with other immune-mediated diseases. BMC Med Genet 7: 20.

5. Belfer I, Buzas B, Hipp H, Dean M, Evans C, et al. (2004) Haplotype structure of inflammatory cytokines genes (*IL1B*, *IL6* and *TNF/LTA*) in US Caucasians and African Americans. Genes Immun 5: 505-512.

6. Conde L, Vaquerizas JM, Dopazo H, Arbiza L, Reumers J, et al. (2006) PupaSuite: finding functional single nucleotide polymorphisms for large-scale genotyping purposes. Nucleic Acids Res 34: W621-625.
